# Cardiac miRNA Expression and their mRNA Targets in a Rat Model of Prediabetes

**DOI:** 10.3390/ijms21062128

**Published:** 2020-03-20

**Authors:** Éva Sághy, Imre Vörös, Bence Ágg, Bernadett Kiss, Gábor Koncsos, Zoltán V. Varga, Anikó Görbe, Zoltán Giricz, Rainer Schulz, Péter Ferdinandy

**Affiliations:** 1 Department of Pharmacology and Pharmacotherapy, Semmelweis University, H-1089 Budapest, Hungary; saghy.eva@med.semmelweis-univ.hu (É.S.); voros.imre@med.semmelweis-univ.hu (I.V.); agg.bence@med.semmelweis-univ.hu (B.Á.); kiss.bernadett@med.semmelweis-univ.hu (B.K.); koncsos.gabor@med.semmelweis-univ.hu (G.K.); varga.zoltan@med.semmelweis-univ.hu (Z.V.V.); gorbe.aniko@med.semmelweis-univ.hu (A.G.); giricz.zoltan@med.semmelweis-univ.hu (Z.G.); 2 MTA-SE System Pharmacology Research Group, Department of Pharmacology and Pharmacotherapy, Semmelweis University, H-1089 Budapest, Hungary; 3 Pharmahungary Group, H-6722 Szeged, Hungary; 4 Heart and Vascular Center, Semmelweis University, H-1122 Budapest, Hungary; 5 HCEMM-SU Cardiometabolic Immunology Research Group, Department of Pharmacology and Pharmacotherapy, Semmelweis University, H-1089 Budapest, Hungary; 6 Institute of Physiology, Justus-Liebig University of Giessen, 35392 Giessen, Germany; Rainer.Schulz@physiologie.med.uni-giessen.de

**Keywords:** prediabetes, heart, network analysis, microRNA, diastolic dysfunction, comorbidities

## Abstract

Little is known about the mechanism of prediabetes-induced cardiac dysfunction. Therefore, we aimed to explore key molecular changes with transcriptomic and bioinformatics approaches in a prediabetes model showing heart failure with preserved ejection fraction phenotype. To induce prediabetes, Long-Evans rats were fed a high-fat diet for 21 weeks and treated with a single low-dose streptozotocin at week 4. Small RNA-sequencing, in silico microRNA (miRNA)-mRNA target prediction, Gene Ontology analysis, and target validation with qRT-PCR were performed in left ventricle samples. From the miRBase-annotated 752 mature miRNA sequences expression of 356 miRNAs was detectable. We identified two upregulated and three downregulated miRNAs in the prediabetic group. We predicted 445 mRNA targets of the five differentially expressed miRNAs and selected 11 mRNAs targeted by three differentially expressed miRNAs, out of which five mRNAs were selected for validation. Out of these five targets, downregulation of three mRNAs i.e., Juxtaposed with another zinc finger protein 1 (*Jazf1*); RAP2C, member of RAS oncogene family (*Rap2c*); and Zinc finger with KRAB and SCAN domains 1 (*Zkscan1*) were validated. This is the first demonstration that prediabetes alters cardiac miRNA expression profile. Predicted targets of differentially expressed miRNAs include *Jazf1*, *Zkscan1*, and *Rap2c* mRNAs. These transcriptomic changes may contribute to the diastolic dysfunction and may serve as drug targets.

## 1. Introduction

Type 2 diabetes mellitus (T2DM) is a common metabolic disorder, the global prevalence of which has been increasing over recent decades [1,2,3]. It is well established that T2DM, a common risk factor of cardiovascular diseases, increases the prevalence and mortality of acute myocardial infarction and heart failure [2,4]. Prediabetes, a precursor state of diabetes mellitus, characterized by impaired glucose tolerance, impaired fasting glucose and raised hemoglobin A1c, increases cardiovascular risk [5]. Early diagnosis of the prediabetic state is important since proper interventions may slow or prevent the progress to diabetes mellitus and reduce the risk of diabetic complications [3,6,7].

It has been reported that prediabetes could induce heart failure with preserved ejection fraction (HFpEF) characterized by diastolic dysfunction in rats [8] and in patients [9,10] where atrial mechanical dysfunction may also be detected. Several molecular mechanisms are shown to contribute to diastolic dysfunction, such as cardiac mitochondrial disturbances, cardiac lipid accumulation, decreased β-myosin heavy chain (*β-MHC*) expression, and decreased Sarcoplasmic/endoplasmic reticulum calcium ATPase 2a (*SERCA2a*) activity [8,11,12,13]. Nevertheless, no effective therapy for the prevention or treatment of HFpEF due to prediabetes is available [8,9,10], plausibly due to our imperfect understanding of the underlying cardiac molecular alterations.

A relatively novel and efficient approach to uncover non-canonical players in pathologies is the use of unbiased microRNA (miRNA)-based transcriptomics methods followed by in silico target prediction by molecular network analysis and experimental target validation [14,15] according to the recommendation of the European Society of Cardiology Working Group on Cellular Biology of the Heart [15]. Although some miRNAs have been previously shown to prevent complications of diabetes mellitus such as cardiomyopathy and nephropathy [16], there are no data in the literature about miRNA-mRNA network changes in the heart due to prediabetes obtained by unbiased methods.

Therefore, the aim of the present study was to explore key molecular changes in a rat model of prediabetes showing HFpEF phenotype with an unbiased approach using small RNA-sequencing, followed by target prediction by molecular network analysis and experimental validation of the predicted molecular targets.

## 2. Results

### 2.1. Differentially Expressed miRNAs

Using small RNA-sequencing analysis, the expression of 356 different miRNAs was detectable in rat left ventricle samples out of the 752 mature miRNA sequences available in the reference annotation [17]. As compared to the control group, rno-miR-141-3p and rno-miR-200c-3p were significantly upregulated while rno-miR-200a-3p, rno-miR-208b-3p and rno-miR-293-5p showed significant downregulation in the prediabetic group (Table 1).

### 2.2. In Silico miRNA Target Prediction and Network Analysis

**Figure 1:**
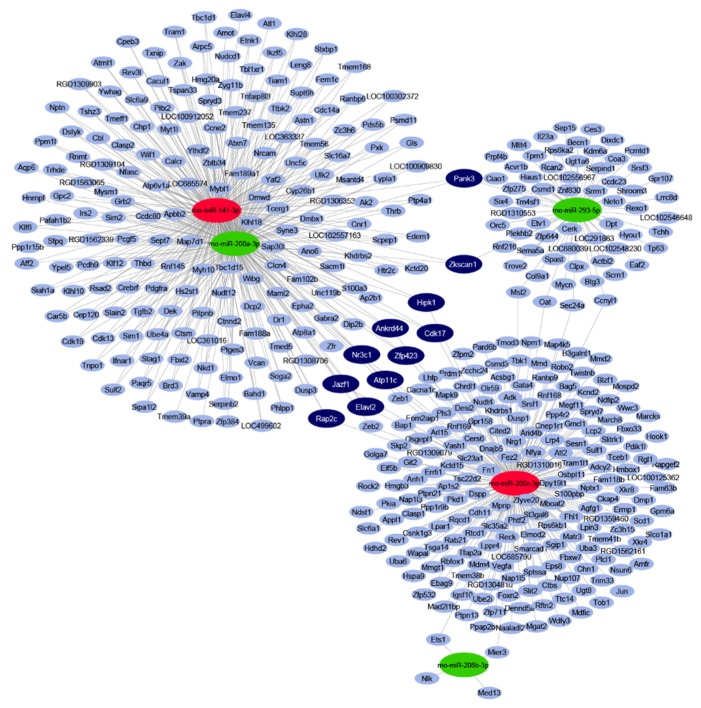
Interaction network and miRNA target prediction analysis of downregulated miRNAs (green) and upregulated miRNAs (red). mRNAs with less than three miRNA interactions are indicated in light-blue colour. mRNAs targeted by three differentially expressed miRNAs are indicated in dark-blue colour.

By in silico miRNA-mRNA target analysis, 445 mRNAs were predicted to be regulated by the five significantly differentially expressed miRNAs. The full list of targets with the interacting miRNAs is shown in Table S1. In the miRNA–target interaction network (Figure 1), the maximal target node degree, i.e., the highest number of interacting miRNAs for one target, was three. Eleven mRNA targets could be characterized by this maximal degree, therefore, these targets were considered to be target hubs with a high probability to be differentially expressed as a result of the miRNA-mediated regulation. All of these 11 targets had a node strength of 1 or -1, meaning that these target hubs were predicted to be regulated by three miRNAs, out of which one had an opposite expression change to the other two (Table S1).

### 2.3. Gene Ontology Analysis of Predicted mRNAs

**Figure 2:**
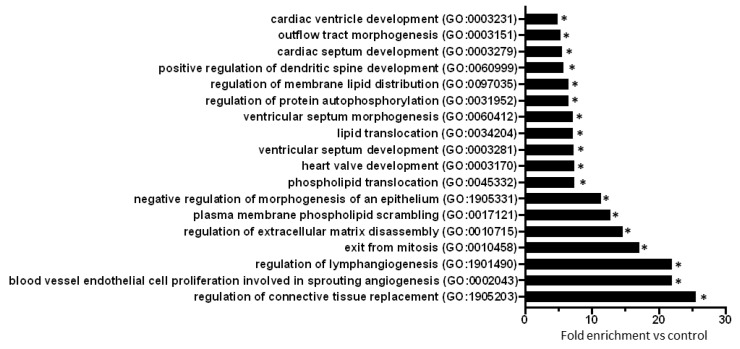
GO analysis (biological processes) of all miRNA target mRNAs (*n* = 445) highlights the effect of prediabetes on cardiac morphogenesis, development, lipid translocation, protein autophosphorylation, connective tissue replacement, extracellular matrix disassembly, and angiogenesis. Eighteen biological processes with the highest fold enrichment value are presented here. *p < 0.001, vs. Control (GO enrichment analysis with Bonferroni correction).

To explore biological processes modified by prediabetes, Gene Ontology (GO) analyses of all predicted mRNAs were performed (Table S2). The result of the GO analysis clearly showed that differentially expressed miRNA-targeted mRNAs were significantly associated with e.g., cardiac morphogenesis, development, lipid translocation, protein autophosphorylation, connective tissue replacement, extracellular matrix disassembly, and angiogenesis-related processes (Figure 2).

### 2.4. Validation of mRNA Targets of Predicted miRNAs

Out of the 11 mRNAs targeted by three differentially expressed miRNAs: Juxtaposed with another zinc finger protein 1 (*Jazf1*); RAP2C, member of RAS oncogene family (*Rap2c*); Zinc finger with KRAB and SCAN domains 1 (*Zkscan1*); Nuclear receptor subfamily 3 group c member 1 (*Nr3c1*); and Pantothenate kinase 3 (*Pank3*) were selected for experimental validation based on the GO analysis (Table 2).

**Figure 3:**
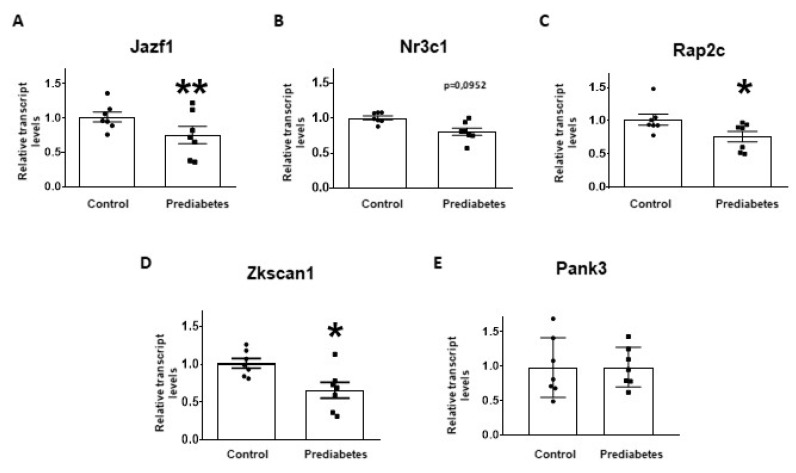
Relative mRNA expression of (**A**) Juxtaposed with another zinc finger protein 1 (*Jazf1*); (**B**) Nuclear receptor subfamily 3 group c member 1 (*Nr3c1*); (**C**) RAP2C, member of RAS oncogene family (*Rap2c*); (**D**) Zinc finger with KRAB and SCAN domains 1 (*Zkscan1*); and (**E**) Pantothenate kinase 3 (*Pank3*) in prediabetic rat left ventricle samples (prediabetes) as compared to vehicle-treated controls (control). The transcript levels were normalized to Hypoxanthine-guanine phosphoribosyltransferase (*HPRT*) mRNA. Data are expressed in arbitrary units as means ± SEM. *n* =7, * *p* < 0.05, ** *p* < 0.01 versus control; unpaired Student’s *t*-test.

To validate the results of small RNA-sequencing and target prediction by molecular network analysis, the five selected mRNA targets were investigated at mRNA level with qRT-PCR. The relative mRNA expression of *Jazf1*, *Rap2c*, and *Zkscan1* significantly decreased in the prediabetic group compared to the control group (Figure 3A,C,D). Although there was a tendency to decrease in the expression of *Nr3c1* in prediabetic animals, this difference was not statistically significant (Figure 3B). We found that the mRNA expression of *Pank3* was not affected by the treatment (Figure 3E).

## 3. Discussion

In the present study, we analysed cardiac miRNA expression and validated their mRNA targets in a rat model of prediabetes induced by high-fat diet and a single low-dose streptozotocin (STZ) treatment. For the first time in the literature, we found that prediabetes induces cardiac miRNA expression changes targeting *Jazf1*, *Zkscan1*, and *Rap2c*. Small RNA-sequencing revealed a cardiac downregulation of rno-miR-200a-3p, rno-miR-293-5p, and rno-miR-208b-3p, and upregulation of rno-miR-141-3p and rno-miR-200c-3p in prediabetes. Out of these five miRNAs, miR-200a-3p, miR-200c-3p, and miR-141-3p belong to the miR-8 family indicating that altered expression of the miR-8 family may play a role in prediabetes showing the HFpEF phenotype. Although some of these miRNAs have been implicated in the pathomechanism of cardiac diseases [18], their connection to the cardiac consequences of prediabetes has not been evidenced.

Here, we have found two upregulated miRNAs, i.e., rno-miR-141-3p and rno-miR-200c-3p, in prediabetic rat hearts. There are some data in the literature concerning these miRNAs in cardiac pathologies. These two miRNAs are transcribed from genes in close proximity on chromosome 4 and thus belong to the same miRNA cluster and to the miR-8 family [17]. Indeed, miR-141-3p and miR-200c-3p often show similar expression patterns in miRNA fingerprint analysis studies [19,20]. In line with our results, the study of Baseler showed that cardiac miR-141 and miR-200c were upregulated in a mouse model of type 1 diabetes mellitus (T1DM) induced by multiple doses of STZ injections [19]. Further supporting our findings, in type 2 diabetic rat hearts induced by a single injection of STZ, microarray analysis revealed the increased expressions of miR-200c and miR-141 [20]. These results show that miR-141-3p and miR-200c-3p may contribute to cardiac pathologies developing in some metabolic disturbances. Previously, we have found in our high-fat, low-dose of STZ prediabetes rat model that gene expression of *β-MHC* decreased in the prediabetic rat heart [8], which correlates with the recently discovered downregulation of miR-208b-3p in our present study. MiR-208b is encoded within the *β-MHC* gene and targets transcriptional repressors of the *β-MHC* [21]. Overexpression of miR-208b, a member of the MyomiR family, is associated with cardiac growth and hypertrophy [22]. Baseler et al. showed the upregulation of not only miR-141 and miR-200c but also miR-208b and miR-295 in type 1 diabetic mouse hearts [19]. MiR-295 belongs to the same miR-290 family as miR-293, identified in our present study. In peripheral blood mononuclear cells of hypertensive patients with HFpEF, a significant increase in miR-208b level was detected compared to hypertensive patients without HFpEF [23]. These results show that miR-208b may contribute to the development of HFpEF. In contrast to miR-208, a limited amount of data has been published on the role of miR-200a-3p and miR-293-5p in cardiac pathologies. In the study of Sun et al., the decrease of miR-200a was observed in tissue samples of patients with end-stage heart failure due to ischemic cardiomyopathy in comparison with non-failing heart samples [24]. Fang L. et al. showed the upregulation of miR-200a-3p in plasma samples of hypertrophic cardiomyopathy patients with diffuse myocardial fibrosis [25].

In the present study, we identified the altered cardiac expression of miRNAs that belong to miR-8, miR-290 and miR-208 miRNA families in prediabetes. According to the studies of Baseler et. al. (2012) and Saito et al. (2016), these families show differential expression in both T1DM and T2DM. However, the total number of differentially expressed miRNA families is higher and both miR-290 and miR-208 families show upregulation in T1DM in contrast to our findings. These results suggest that prior to the development of advanced diabetes, some of the differentially expressed miRNAs or miRNA families appear even in prediabetes.

Here, we performed miRNA-mRNA target prediction by molecular network analysis to explore mRNA targets of the differentially expressed miRNAs. We validated five mRNA targets out of 445 mRNAs interacting with miRNAs with altered expression. *Jazf1* is a zinc finger protein and an inhibitor of the Nuclear receptor subfamily 2, group C, member 2 (*Nr2c2*), which is involved in insulin resistance, glucose and lipid metabolism, and regulation of peroxisome proliferator-activated receptor (*PPAR*)α, β/δ, γ [26,27]. Interestingly, *Jazf1* was found to be downregulated in pancreatic islets from T2DM patients, and increased expression of *Jazf1* was associated with higher insulin secretion and lower hemoglobin A1c [28]. Moreover, Ho et al. (2013) suggested that decreased expression of *Jazf1* may be a consequence of hyperglycemia [29]. Thus, *Jazf1* may serve as a target for future therapies of consequences of metabolic derangements. Here we also found *Rap2c* (a member of the Ras-related protein subfamily of the Ras GTPase superfamily) and *Zkscan1* (encodes a member of the Kruppel C2H2-type zinc-finger family of proteins) expression to be downregulated in the prediabetic rat heart. Although Shaum et al. showed that *Zkscan1* and *Rap2c* are expressed in most of the cell types of mouse heart [30], their function remains unclear.

Although here we successfully validated the predicted mRNA targets of differentially expressed miRNAs by qRT-PCR, there are three major limitations of our present study. 1. The direct interactions of miRNA–mRNA were not validated experimentally, however, bioinformatics prediction is a powerful evidence for microRNA–target interaction since mirRNAtarget^TM^ integrates miRDB, microRNA.org and also the experimentally validated miRTarBase database [14,31,32]. 2. Validation of the predicted mRNA targets at protein level was out of the scope of our present study. 3. The function of the newly identified targets was not validated either pharmacologically due to the lack of specific pharmacological inhibitors or by genetic approaches (gene silencing, overexpression) due to the need for extensive further experimentation which was outside the scope of this study.

## 4. Materials and Methods

### 4.1. Characterization of Prediabetes Model and Tissue Sampling

Control and prediabetic heart samples of the present study have been obtained from our previous study Koncsos et al. (2016) [8]. Sample preparation, prediabetes model protocol, and metabolic and cardiac parameters were described in the study of Koncsos et al. (2016) in detail [8]. Briefly, to induce prediabetes, Long-Evans rats were fed a high-fat (40%) chow for 21 weeks and treated with a single low dose (20 mg/kg) of STZ at week 4. To produce high-fat diet chow we added commercially available lard (40%) to the standard chow (ssniff Spezialdiäten GmbH, Soest, Germany). Fatty acid components of the high fat chow include: oleic acid (40.78%), palmitic acid (23.02%), stearic acid (16.00%), linoleic acid (11.81%), octadecenoic acid isomer B (2.85%), palmitoleic acid (1.59%), and others (altogether less than 4%). Control animals were fed a standard chow for 21 weeks and treated with the vehicle of STZ at week 4 [8]. In the study of Koncsos et al. [8], we demonstrated that high-fat and STZ treatment induced prediabetes that is characterized by a slight elevation in fasting blood glucose, impaired glucose and insulin tolerance and an increase in visceral adipose tissue, leading to diastolic dysfunction and cardiac hypertrophy. At week 21, hearts were excised under pentobarbital (60 mg/kg, i.p.) anaesthesia and perfused with oxygenated Krebs-Henseleit solution in Langendorff mode and stored at −80°C in RNAlater [8].

### 4.2. RNA Isolation and Small RNA-Sequencing

RNAlater-preserved rat left ventricle samples (*n* = 6/group) were thawed on ice, then the left ventricular tissue was placed in a 1.5 mL Eppendorf LoBind tube containing glass beads (1.7–2.1 mm diameter, Carl Roth, Karlsruhe, Germany) and 500 µL of VRX buffer (Viogene Biotek, New Taipei City, Taiwan, R.O.C). The Eppendorf tube was firmly attached to a SILAMAT S5 vibrator (Ivoclar Vivadent, Schaan, Liechtenstein) in order to disrupt and homogenize the tissue for 15 s. Total RNA was extracted using Viogene miTotal RNA Extraction Miniprep System (Viogene Biotek, New Taipei City, Taiwan, R.O.C) according to the manufacturer’s protocol. The RNA Integrity Numbers and RNA concentration were determined by the RNA ScreenTape system with 2200 Tapestation (Agilent Technologies, Santa Clara, CA, USA) and the RNA HS Assay Kit with Qubit 3.0 Fluorometer (Thermo Fisher Scientific, Waltham, MA, USA), respectively.

For small RNA library construction, the NEBNext Multiplex Small RNA Library Prep Set for Illumina (New England Biolabs, Ipswich, MA, USA) was applied according to the manufacturer’s protocol. The quality and quantity of the library QC was performed by using the High Sensitivity DNA1000 ScreenTape system with 2200 Tapestation (Agilent Technologies, Santa Clara, CA, USA) and dsDNA HS Assay Kit with Qubit 3.0 Fluorometer (Thermo Fisher Scientific, Waltham, MA, USA), respectively. Pooled libraries were diluted to 1.8 pM for 2 × 43 bp paired-end sequencing with 75-cycle High Output v2 Kit on the NextSeq 550 Sequencing System (Illumina, San Diego, CA, USA) at the Xenovea Ltd. according to the manufacturer’s protocol. Small RNA-sequencing datasets were stored in the ArrayExpress database (https://www.ebi.ac.uk/arrayexpress/ with accession number: E-MTAB-8204).

### 4.3. Bioinformatics Analysis of Small RNA-Sequencing Data

After adapter trimming, quality and length filtering of the raw sequencing reads by Illumina FastQ Toolkit (version v2.2.0) only reads with an average Phred quality score over 30 and with a length of at least 10 nt were kept for further analysis. Following quality control analysis by FastQC (version v0.11.7), alignment of the filtered reads to Rnor_6.0 NCBI *Rattus norvegicus* reference genome assembly was performed by Bowtie 2 aligner (version 2.2.1) [33]. Reads mapped to mature miRNA loci were counted by the featureCounts software (version v1.6.2) [34] using the miRBase release 22.1 *Rattus norvegicus* reference annotation [17]. EdgeR (version 3.12.1) [35] Bioconductor package was utilized for normalization and differential expression analysis with likelihood ratio tests after applying GLM to estimate dispersions. MiRNAs were considered to be significantly differentially expressed if the FDR (corrected *p*-value according to Benjamini and Hochberg) [36] remained under 0.05.

### 4.4. miRNA Target Prediction and miRNA-mRNA Target Network Analysis

To predict likely targets of differentially expressed miRNAs and their combined effect on their common targets, a miRNA–target interaction network was constructed by the miRNAtarget™ software (https://mirnatarget.com; Pharmahungary, Szeged, Hungary) as described previously [14,31]. Both predicted (miRDB version 5.0 with score > 80.0 and microRNA.org with mirSVR score < −1.2) and experimentally validated, manually curated (miRTarBase 4.5) miRNA–target interactions were integrated by miRNAtarget™ [37,38,39]. In the resulting network, miRNAs and their targets were represented by nodes and were connected by edges of predicted interactions. After assigning positive or negative weights (1 or -1) to edges based on the up- or downregulation of the interacting miRNA, respectively, node strength was calculated for each target node by summing the incoming edge weights.

Optimal network visualization was obtained by the EntOptLayout plugin (version 2.1) for the Cytoscape (version 3.6) network analysis framework using double consideration of the main diagonal of the adjacency matrix and sequential position and weight updates achieving a high quality layout with negligible normalized information loss of 0.027 [40].

### 4.5. Gene Ontology Analysis

GO analysis using all predicted miRNA target mRNAs was conducted with the online tool of Gene Ontology Consortium (geneontology.org). *Rattus norvegicus* species annotations were adopted from the PANTHER Classification System (pantherdb.org) on 17 June 2019. GO enrichment analysis with FDR correction was performed [41,42].

### 4.6. Selection of miRNA Target mRNAs for Experimental Validation

Out of the predicted 11 miRNA target hubs (i.e., mRNAs regulated by three miRNAs), two targets had a node strength of -1 and 9 targets had a node strength of +1. All miRNA target hubs with a node strength of -1 and those three targets with a +1 node strength and annotated with the biological processes having the highest fold enrichment value based on GO analysis were selected for experimental validation (Figure 2).

### 4.7. RNA Isolation and qRT-PCR

The total RNA was isolated from control and prediabetic left ventricle samples with Direct-zol RNA MiniPrep according to the manufacturer’s instructions (Zymo Research Corporation Safety Department, Irvine, CA, USA). Afterwards, cDNA was synthesized from total RNA applying Sensifast cDNA synthesis kit (Bioline, London, UK) according to the manufacturer’s protocol. To detect the transcript levels of *Jazf1*, *Nr3c1*, *Rap2c*, *Zkscan1*, and *Pank3*, quantitative real-time polymerase chain reaction (qRT-PCR) was performed with SensiFAST SYBR No-ROX mix (SensiFAST^TM^ SYBR^®^ No-ROX kit, Bioline, London, UK) on a Light Cycler^®^ 480 (Roche, Applied Science, Penzberg, Upper Bavaria, Germany). Hypoxanthine-guanine phosphoribosyltransferase (*HPRT*) was used as a reference gene. Primer pairs for *Jazf1*, *Nr3c1*, *Rap2c*, *Zkscan1*, *Pank3*, and *HPRT* were designed against sequences of intron-spanning exons by Primer-BLAST software (http://www.ncbi.nlm.nih.gov/tools/primer-blast/) and tested to avoid primer dimers, non-specific amplification, and self-priming by this software (Table 3).

Cycle conditions were the following: 95 °C for 2 min, followed by 40 cycles of 95 °C for 5 s, 61 °C for 10 s, then 72 °C for 20 s. Every reaction was implemented in duplicate to ensure the reliability of single values. Measurements included dissociation curve analysis and amplicon length verification on 2% agarose gel to ensure amplification specificity. 2^−ΔΔ*C*p^ methods were used to determine the relative gene expression ratio.

### 4.8. Statistical Analysis

Unpaired Student’s *t*-test was performed to compare the data of qRT-PCR analysis with GraphPad Prism 8.0.1. (GraphPad Software Inc.; San Diego, CA, USA). Data are shown as mean ± standard error of mean (SEM), *p* < 0.05 was considered statistically significant.

## 5. Conclusions

In conclusion, our present results show that mild prediabetes induces miRNA expression changes leading to altered gene expression of *Jazf1*, *Zkscan1*, and *Rap2c*, which may contribute to the diastolic dysfunction and may serve as potential drug targets. However, the mechanistic role of the identified molecular targets in prediabetes-induced HFpEF should be further investigated.

## Figures and Tables

**Table 1:** Differentially expressed miRNAs from left ventricular samples. LogFC, base 2 logarithm of the fold change; *p*-values were calculated by the edgeR software package using likelihood ratio tests after applying generalized linear models (GLM) to estimate dispersions. FDR, false-discovery rate (adjusted *p*-value according to Benjamini and Hochberg).

miRNA Name	logFC	*p*-Value	FDR	Expression Change
rno-miR-141-3p	2.49	< 0.001	< 0.001	up
rno-miR-200a-3p	−1.41	< 0.001	0.037	down
rno-miR-200c-3p	2.51	< 0.001	< 0.001	up
rno-miR-208b-3p	−1.56	< 0.001	0.012	down
rno-miR-293-5p	−1.99	0.001	0.045	down

**Table 2:** Selected target genes indicating their miRNA connections. ↓ indicates downregulation and ↑ indicates upregulation of the selected gene targets in the prediabetic group as compared to those in the control group, + indicates predicted interaction between the selected target genes and the differentially expressed miRNAs.

Target		Predicted to be Regulated by			
Abbreviation	Name	*miR-141-3p ↑*	miR-200a-3p ↓	*miR-200c-3p ↑*	miR-293-5p ↓
*Nr3c1*	Nuclear receptor subfamily 3 group c member 1	+	+	+	
*Jazf1*	Juxtaposed with another zinc finger protein 1	+	+	+	
*Rap2c*	RAP2C, member of RAS oncogene family	+	+	+	
*Zkscan1*	Zinc finger with KRAB and SCAN domains 1	+	+		+
*Pank3*	Pantothenate kinase 3	+	+		+

**Table 3:** Primer properties used in qRT-PCR for the determination of transcript levels. *Nr3c1*, nuclear receptor subfamily 3 group c member 1; *Jazf1*, Jazf zinc finger protein 1; *Rap2c*, member of RAS oncogene family; *Zkscan1*, zinc finger with KRAB and SCAN domains 1; *Pank3*, Pantothenate kinase 3; *HPRT*, Hypoxanthine-guanine phosphoribosyltransferase; and bp, base pair.

Target	Accession Number	Forward Primer	Reverse Primer	Product Size (bp)
*Nr3c1*	NM_012576.2	AGGCGATACCAGGCTTCAGA	TCAGGAGCAAAGCAGAGCAG	142
*Jazf1*	XM_001065610.6	CCAACAGGCAGCGAGTATGA	AGGCTTCTCTTCCCCTCCAT	138
*Rap2c*	NM_001106950.2	GGCCATACCGAGCAGATAAAAAC	TGGATCTGGAGGGCCAAAGA	164
*Zkscan1*	NM_001025760.1	GGAGTCCTCAAGCTTCGACC	GATCTTCACCATTGCCTGGGA	193
*Pank3*	NM_001108272.2	TGGGCTGTGGCATCTAGTTTT	AACAGCACACATTCGAGCCA	135
*HPRT*	NM_012583.2	GTCCTGTTGATGTGGCCAGT	TGCAAATCAAAAGGGACGCA	144

## Supplementary Materials

The following are available online at https://www.mdpi.com/1422-0067/21/6/2128/s1, Table S1: Predicted miRNA-target interaction list, Table S2: Detailed results of Gene Ontology enrichment analysis of the predicted targets of differentially expressed miRNAs.

## Abbreviations

Cp	crossing point values
FDR	false-discovery rate
GLM	generalized linear models
GO	Gene Ontology
HFpEF	heart failure with preserved ejection fraction
*HPRT*	Hypoxanthine-guanine phosphoribosyltransferase
*Jazf1*	Juxtaposed with another zinc finger protein 1
miRNA	microRNA
*Nr2c2*	Nuclear receptor subfamily 2 group c member 2
*Nr3c1*	Nuclear receptor subfamily 3 group c member 1
*Pank3*	Pantothenate kinase 3
*PPAR*	peroxisome proliferator-activated receptor
*Rap2c*	RAP2C, member of RAS oncogene family
qRT-PCR	quantitative real-time polymerase chain reaction
*SERCA*	Sarcoplasmic/endoplasmic reticulum calcium ATPase
STZ	streptozotocin
T1DM	type 1 diabetes mellitus
T2DM	type 2 diabetes mellitus
*Zkscan1*	Zinc finger protein with KRAB and SCAN domains 1
*β-MHC*	β-myosin heavy chain
