# Supplementary material for: Cardiac miRNA Expression and their mRNA Targets in a Rat Model of Prediabetes
Source: Int J Mol Sci. 2020 Mar 20;21(6):2128. doi: 10.3390/ijms21062128 (PMC7139428; doi:10.3390/ijms21062128)
Supplement: Supplementary file 1 [file ijms-21-02128-s001.zip › sTable 2.pdf]

#	GO Biological process	Expected count	Input count	Fold enrichment	FDR
		Target gene symbol	Target gene name	Target node strentgth	Interacting miRNA list
1	regulation of connective tissue replacement	0.12	3	25.55	5.41E-04
		Rock2	Rho-associated protein kinase 2	1	rno-miR-200c;
		Gata4	Transcription factor GATA-4	1	rno-miR-200c;
		Klf6	Krueppel-like factor 6	0	rno-miR-141;rno-miR-200a;
2	blood vessel endothelial cell proliferation involved in sprouting angiogenesis	0.14	3	21.90	7.62E-04
		Vegfa	Vascular endothelial growth factor A	1	rno-miR-200c;
		Sema5a	Semaphorin-5A	-1	rno-miR-293;
		Epha2	Eph receptor A2	0	rno-miR-141;rno-miR-200a;
3	regulation of lymphangiogenesis	0.14	3	21.90	7.62E-04
		Vegfa	Vascular endothelial growth factor A	1	rno-miR-200c;
		Vash1	Vasohibin 1	1	rno-miR-200c;
		Epha2	Eph receptor A2	0	rno-miR-141;rno-miR-200a;
4	exit from mitosis	0.23	4	17.04	2.03E-04
		Clasp1	Cytoplasmic linker-associated protein 1	1	rno-miR-200c;
		Spast	Spastin	-1	rno-miR-293;
		Eps8	Epidermal growth factor receptor kinase substrate 8	1	rno-miR-200c;
		Clasp2	CLIP-associating protein 2	0	rno-miR-141;rno-miR-200a;
5	regulation of extracellular matrix disassembly	0.27	4	14.60	3.31E-04
		Clasp1	Cytoplasmic linker-associated protein 1	1	rno-miR-200c;
		Ets1	Protein C-ets-1	0	rno-miR-200c;rno-miR-208b-3p;
		Tgfb2	Transforming growth factor beta-2	0	rno-miR-141;rno-miR-200a;
		Clasp2	CLIP-associating protein 2	0	rno-miR-141;rno-miR-200a;
6	plasma membrane phospholipid scrambling	0.31	4	12.78	5.08E-04
		Pls3	Phospholipid scramblase 3	1	rno-miR-200c;
		Xkr8	XK-related protein	1	rno-miR-200c;
		Xkr4	XK-related protein 4	1	rno-miR-200c;
		Ano6	Anoctamin	0	rno-miR-141;rno-miR-200a;
7	negative regulation of morphogenesis of an epithelium	0.35	4	11.36	7.44E-04
		Clasp1	Cytoplasmic linker-associated protein 1	1	rno-miR-200c;
		Sulf1	Extracellular sulfatase Sulf-1	1	rno-miR-200c;
		Nkd1	Naked cuticle homolog 1	0	rno-miR-141;rno-miR-200a;
		Clasp2	CLIP-associating protein 2	0	rno-miR-141;rno-miR-200a;
8	heart valve development	1.10	8	7.30	2.98E-05
		Robo2	Roundabout guidance receptor 2	1	rno-miR-200c;
		Slit2	Slit homolog 2 protein (Fragment)	1	rno-miR-200c;
		Prdm1	PR domain containing 1, with ZNF domain (Predicted)	1	rno-miR-200c;
		Mdm4	Protein Mdm4	1	rno-miR-200c;
		Gata4	Transcription factor GATA-4	1	rno-miR-200c;
		Matr3	Matrin-3	1	rno-miR-200c;
		Tgfb2	Transforming growth factor beta-2	0	rno-miR-141;rno-miR-200a;
		Pitx2	Pituitary homeobox 2	0	rno-miR-141;rno-miR-200a;
9	ventricular septum morphogenesis	0.84	6	7.13	3.35E-04
		Robo2	Roundabout guidance receptor 2	1	rno-miR-200c;
		Slit2	Slit homolog 2 protein (Fragment)	1	rno-miR-200c;
		Cited2	Cbp/p300-interacting transactivator, with Glu/Asp-rich carboxy-terminal domain, 2	1	rno-miR-200c;
		Zfpm2	Zinc finger protein, multitype 2	1	rno-miR-200c;
		Tgfb2	Transforming growth factor beta-2	0	rno-miR-141;rno-miR-200a;
		Pitx2	Pituitary homeobox 2	0	rno-miR-141;rno-miR-200a;
10	regulation of protein autophosphorylation	0.92	6	6.52	5.16E-04
		Rap2c	RAP2C, member of RAS oncogene family	1	rno-miR-141;rno-miR-200a;rno-miR-200c;
		Vegfa	Vascular endothelial growth factor A	1	rno-miR-200c;
		Errfi1	ERBB receptor feedback inhibitor 1	1	rno-miR-200c;
		Nrg1	Pro-neuregulin-1, membrane-bound isoform	1	rno-miR-200c;
		Jun	Transcription factor AP-1	1	rno-miR-200c;
		Chp1	Calcineurin B homologous protein 1	0	rno-miR-141;rno-miR-200a;
11	positive regulation of dendritic spine development	1.21	7	5.77	3.53E-04
		Lpar1	Lysophosphatidic acid receptor 1	1	rno-miR-200c;
		Nrg1	Pro-neuregulin-1, membrane-bound isoform	1	rno-miR-200c;
		Marcks	Myristoylated alanine-rich C-kinase substrate	1	rno-miR-200c;
		Mllt4	Afadin	-1	rno-miR-293;
		Nr3c1	Glucocorticoid receptor	1	rno-miR-141;rno-miR-200a;rno-miR-200c;
		Cpeb3	Cytoplasmic polyadenylation element-binding protein 3	0	rno-miR-141;rno-miR-200a;
		Tiam1	T-cell lymphoma invasion and metastasis 1	0	rno-miR-141;rno-miR-200a;
12	outflow tract morphogenesis	1.51	8	5.31	2.28E-04
		Robo2	Roundabout guidance receptor 2	1	rno-miR-200c;
		Cited2	Cbp/p300-interacting transactivator, with Glu/Asp-rich carboxy-terminal domain, 2	1	rno-miR-200c;
		Zfpm2	Zinc finger protein, multitype 2	1	rno-miR-200c;
		Vegfa	Vascular endothelial growth factor A	1	rno-miR-200c;
		Tfap2a	Transcription factor AP-2-alpha	1	rno-miR-200c;
		Jun	Transcription factor AP-1	1	rno-miR-200c;
		Tgfb2	Transforming growth factor beta-2	0	rno-miR-141;rno-miR-200a;
		Pitx2	Pituitary homeobox 2	0	rno-miR-141;rno-miR-200a;
13	regulation of protein depolymerization	1.70	8	4.70	4.88E-04
		Clasp1	Cytoplasmic linker-associated protein 1	1	rno-miR-200c;
		Tpm1	Tropomyosin alpha-1 chain	-1	rno-miR-293;
		Sema5a	Semaphorin-5A	-1	rno-miR-293;
		Spast	Spastin	-1	rno-miR-293;
		Eps8	Epidermal growth factor receptor kinase substrate 8	1	rno-miR-200c;
		Tmod3	RCG25684, isoform CRA_a	1	rno-miR-200c;
		Ttbk2	Tau tubulin kinase 2	0	rno-miR-141;rno-miR-200a;
		Clasp2	CLIP-associating protein 2	0	rno-miR-141;rno-miR-200a;
14	positive regulation of axonogenesis	2.00	9	4.51	2.92E-04
		Robo2	Roundabout guidance receptor 2	1	rno-miR-200c;
		Vegfa	Vascular endothelial growth factor A	1	rno-miR-200c;
		Dixdc1	Dixin	-1	rno-miR-293;
		Sema5a	Semaphorin-5A	-1	rno-miR-293;
		Nrg1	Pro-neuregulin-1, membrane-bound isoform	1	rno-miR-200c;
		Slitrk1	SLIT and NTRK-like family, member 1	1	rno-miR-200c;
		Fn1	Fibronectin	1	rno-miR-200c;
		Tiam1	T-cell lymphoma invasion and metastasis 1	0	rno-miR-141;rno-miR-200a;
		Zeb2	Zinc finger E-box-binding homeobox 2	0	rno-miR-200a;rno-miR-200c;
15	lung development	4.68	18	3.85	2.66E-06
		Zfpm2	Zinc finger protein, multitype 2	1	rno-miR-200c;
		Mycn	N-myc proto-oncogene protein	-1	rno-miR-293;
		Vegfa	Vascular endothelial growth factor A	1	rno-miR-200c;
		Tmem38b	Trimeric intracellular cation channel type B	1	rno-miR-200c;
		Pkd1	Polycystin 1, transient receptor potential channel-interacting	1	rno-miR-200c;
		Fbxw7	F-box/WD repeat-containing protein 7	1	rno-miR-200c;
		Dspp	Dentin sialophosphoprotein	1	rno-miR-200c;
		Gata4	Transcription factor GATA-4	1	rno-miR-200c;
		Errfi1	ERBB receptor feedback inhibitor 1	1	rno-miR-200c;
		Slc23a1	Solute carrier family 23 member 1	1	rno-miR-200c;
		Nr3c1	Glucocorticoid receptor	1	rno-miR-141;rno-miR-200a;rno-miR-200c;
		Pdgfra	Platelet-derived growth factor receptor alpha	0	rno-miR-141;rno-miR-200a;
		Tshz3	Teashirt homolog 3	0	rno-miR-141;rno-miR-200a;
		Thrb	Thyroid hormone receptor beta	0	rno-miR-141;rno-miR-200a;
		Sim2	Single-minded 2 (Predicted)	0	rno-miR-141;rno-miR-200a;
		Ptges3	Prostaglandin E synthase 3	0	rno-miR-141;rno-miR-200a;
		Tgfb2	Transforming growth factor beta-2	0	rno-miR-141;rno-miR-200a;
		Pitx2	Pituitary homeobox 2	0	rno-miR-141;rno-miR-200a;
16	regulation of cell shape	2.72	10	3.68	6.23E-04
		Lpar1	Lysophosphatidic acid receptor 1	1	rno-miR-200c;
		Vegfa	Vascular endothelial growth factor A	1	rno-miR-200c;
		Tpm1	Tropomyosin alpha-1 chain	-1	rno-miR-293;
		Eps8	Epidermal growth factor receptor kinase substrate 8	1	rno-miR-200c;
		Shroom3	Shroom family member 3	-1	rno-miR-293;
		Fn1	Fibronectin	1	rno-miR-200c;
		Thrb	Prothrombin	0	rno-miR-141;rno-miR-200a;
		Syne3	Spectrin repeat-containing, nuclear envelope family member 3	0	rno-miR-141;rno-miR-200a;
		Myh10	Myosin-10	0	rno-miR-141;rno-miR-200a;
		Sept7	Septin-7	0	rno-miR-141;rno-miR-200a;
17	development of primary female sexual characteristics	2.76	10	3.62	6.91E-04
		Robo2	Roundabout guidance receptor 2	1	rno-miR-200c;
		Slit2	Slit homolog 2 protein (Fragment)	1	rno-miR-200c;
		Vegfa	Vascular endothelial growth factor A	1	rno-miR-200c;
		Acvr1b	Activin receptor type-1B	-1	rno-miR-293;
		Nup107	Nuclear pore complex protein Nup107	1	rno-miR-200c;
		Ermp1	Endoplasmic reticulum metallopeptidase 1	1	rno-miR-200c;
		Acsbg1	Long-chain-fatty-acid--CoA ligase ACSBG1	1	rno-miR-200c;
		Znf830	Zinc finger protein 830	-1	rno-miR-293;
		Pdgfra	Platelet-derived growth factor receptor alpha	0	rno-miR-141;rno-miR-200a;
		Pitx2	Pituitary homeobox 2	0	rno-miR-141;rno-miR-200a;
18	positive regulation of epithelial cell migration	2.80	10	3.57	7.65E-04

		Rock2	Rho-associated protein kinase 2	1	rno-miR-200c;
		Clasp1	Cytoplasmic linker-associated protein 1	1	rno-miR-200c;
		Vegfa	Vascular endothelial growth factor A	1	rno-miR-200c;
		Sema5a	Semaphorin-5A	-1	rno-miR-293;
		Ppap2b	Phospholipid phosphatase 3	1	rno-miR-200c;
		Jun	Transcription factor AP-1	1	rno-miR-200c;
		Amot	Angiomotin	1	rno-miR-141;
		Ets1	Protein C-ets-1	0	rno-miR-200c;rno-miR-208b-3p;
		Tgfb2	Transforming growth factor beta-2	0	rno-miR-141;rno-miR-200a;
		Clasp2	CLIP-associating protein 2	0	rno-miR-141;rno-miR-200a;
19	axon guidance	3.95	14	3.54	7.93E-05
		Robo2	Roundabout guidance receptor 2	1	rno-miR-200c;
		Slit2	Slit homolog 2 protein (Fragment)	1	rno-miR-200c;
		Vegfa	Vascular endothelial growth factor A	1	rno-miR-200c;
		Sema5a	Semaphorin-5A	-1	rno-miR-293;
		Chn1	N-chimaerin	1	rno-miR-200c;
		Etv1	Ets variant 1	-1	rno-miR-293;
		Apbb2	Amyloid beta precursor protein-binding family B member 2	0	rno-miR-141;rno-miR-200a;
		Nfasc	Neurofascin	0	rno-miR-141;rno-miR-200a;
		Unc5c	Netrin receptor UNC5C	0	rno-miR-141;rno-miR-200a;
		Nrcam	Neuronal cell adhesion molecule	0	rno-miR-141;rno-miR-200a;
		Nptn	Neuroplastin	0	rno-miR-141;rno-miR-200a;
		Myh10	Myosin-10	0	rno-miR-141;rno-miR-200a;
		Tgfb2	Transforming growth factor beta-2	0	rno-miR-141;rno-miR-200a;
		Epha2	Eph receptor A2	0	rno-miR-141;rno-miR-200a;
20	embryonic limb morphogenesis	2.84	10	3.52	8.46E-04
		Mycn	N-myc proto-oncogene protein	-1	rno-miR-293;
		Lrp4	Low-density lipoprotein receptor-related protein 4	1	rno-miR-200c;
		Tfap2a	Transcription factor AP-2-alpha	1	rno-miR-200c;
		Cacna1c	Voltage-dependent L-type calcium channel subunit alpha-1C	1	rno-miR-200c;
		Tp63	Tumor protein 63	-1	rno-miR-293;
		Reck	RCG54895, isoform CRA_a	1	rno-miR-200c;
		Ttbk2	Tau tubulin kinase 2	0	rno-miR-141;rno-miR-200a;
		Tgfb2	Transforming growth factor beta-2	0	rno-miR-141;rno-miR-200a;
		Pitx2	Pituitary homeobox 2	0	rno-miR-141;rno-miR-200a;
		Cyp26b1	Cytochrome P450 26B1	0	rno-miR-141;rno-miR-200a;
21	stem cell differentiation	3.21	11	3.43	5.88E-04
		Cited2	Cbp/p300-interacting transactivator, with Glu/Asp-rich carboxy-terminal domain, 2	1	rno-miR-200c;
		Sema5a	Semaphorin-5A	-1	rno-miR-293;
		Tfap2a	Transcription factor AP-2-alpha	1	rno-miR-200c;
		Nrg1	Pro-neuregulin-1, membrane-bound isoform	1	rno-miR-200c;
		Tp63	Tumor protein 63	-1	rno-miR-293;
		Fn1	Fibronectin	1	rno-miR-200c;
		Gpm6a	Neuronal membrane glycoprotein M6-a	1	rno-miR-200c;
		Psmd11	26S proteasome non-ATPase regulatory subunit 11	0	rno-miR-141;rno-miR-200a;
		Tgfb2	Transforming growth factor beta-2	0	rno-miR-141;rno-miR-200a;
		Pitx2	Pituitary homeobox 2	0	rno-miR-141;rno-miR-200a;
		Zeb2	Zinc finger E-box-binding homeobox 2	0	rno-miR-200a;rno-miR-200c;
22	response to transforming growth factor beta	3.27	11	3.37	6.77E-04
		Cited2	Cbp/p300-interacting transactivator, with Glu/Asp-rich carboxy-terminal domain, 2	1	rno-miR-200c;
		Rock2	Rho-associated protein kinase 2	1	rno-miR-200c;
		Acvr1b	Activin receptor type-1B	-1	rno-miR-293;
		Dspp	Dentin sialophosphoprotein	1	rno-miR-200c;
		Nlk	Serine/threonine-protein kinase NLK	-1	rno-miR-208b-3p;
		Appl1	Adaptor protein, phosphotyrosine-interacting with PH domain and leucine zipper 1	1	rno-miR-200c;
		Jun	Transcription factor AP-1	1	rno-miR-200c;
		Fn1	Fibronectin	1	rno-miR-200c;
		Nr3c1	Glucocorticoid receptor	1	rno-miR-141;rno-miR-200a;rno-miR-200c;
		Tgfb2	Transforming growth factor beta-2	0	rno-miR-141;rno-miR-200a;
		Zeb1	Zinc finger E-box-binding homeobox 1	0	rno-miR-200a;rno-miR-200c;
23	protein localization to plasma membrane	3.64	12	3.30	4.67E-04
		Rock2	Rho-associated protein kinase 2	1	rno-miR-200c;
		Mllt4	Afadin	-1	rno-miR-293;
		Rapgef2	Rap guanine nucleotide exchange factor 2	1	rno-miR-200c;
		Gpr158	Probable G-protein coupled receptor 158	1	rno-miR-200c;
		Blzf1	Basic leucine zipper nuclear factor 1	1	rno-miR-200c;
		Golga7	Golgin subfamily A member 7	1	rno-miR-200c;
		Nfasc	Neurofascin	0	rno-miR-141;rno-miR-200a;
		Stxbp1	Syntaxin-binding protein 1	0	rno-miR-141;rno-miR-200a;
		Tspan33	Tetraspanin	0	rno-miR-141;rno-miR-200a;
		Vamp4	Vesicle-associated membrane protein 4	0	rno-miR-141;rno-miR-200a;
		Clasp2	CLIP-associating protein 2	0	rno-miR-141;rno-miR-200a;
		Epha2	Eph receptor A2	0	rno-miR-141;rno-miR-200a;
24	regulation of animal organ morphogenesis	4.09	13	3.18	3.82E-04
		Robo2	Roundabout guidance receptor 2	1	rno-miR-200c;
		Cited2	Cbp/p300-interacting transactivator, with Glu/Asp-rich carboxy-terminal domain, 2	1	rno-miR-200c;
		Vegfa	Vascular endothelial growth factor A	1	rno-miR-200c;
		Dspp	Dentin sialophosphoprotein	1	rno-miR-200c;
		Dmp1	Dentin matrix acidic phosphoprotein 1	1	rno-miR-200c;
		Tfap2a	Transcription factor AP-2-alpha	1	rno-miR-200c;
		Sulf1	Extracellular sulfatase Sulf-1	1	rno-miR-200c;
		Six4	SIX homeobox 4	-1	rno-miR-293;
		Pdgfra	Platelet-derived growth factor receptor alpha	0	rno-miR-141;rno-miR-200a;
		Thrb	Thyroid hormone receptor beta	0	rno-miR-141;rno-miR-200a;
		Nkd1	Naked cuticle homolog 1	0	rno-miR-141;rno-miR-200a;
		Tgfb2	Transforming growth factor beta-2	0	rno-miR-141;rno-miR-200a;
		Sulf2	Extracellular sulfatase	0	rno-miR-141;rno-miR-200a;
25	extracellular matrix organization	4.50	14	3.11	2.84E-04
		Col9a1	Collagen alpha-1(IX) chain (Fragment)	-1	rno-miR-293;
		Tmem38b	Trimeric intracellular cation channel type B	1	rno-miR-200c;
		Dspp	Dentin sialophosphoprotein	1	rno-miR-200c;
		Dmp1	Dentin matrix acidic phosphoprotein 1	1	rno-miR-200c;
		Tfap2a	Transcription factor AP-2-alpha	1	rno-miR-200c;
		Sulf1	Extracellular sulfatase Sulf-1	1	rno-miR-200c;
		Reck	RCG54895, isoform CRA_a	1	rno-miR-200c;
		Dpt	Dermatopontin	-1	rno-miR-293;
		Fn1	Fibronectin	1	rno-miR-200c;
		Apbb2	Amyloid beta precursor protein-binding family B member 2	0	rno-miR-141;rno-miR-200a;
		Pdgfra	Platelet-derived growth factor receptor alpha	0	rno-miR-141;rno-miR-200a;
		Tgfb2	Transforming growth factor beta-2	0	rno-miR-141;rno-miR-200a;
		Sulf2	Extracellular sulfatase	0	rno-miR-141;rno-miR-200a;
		Ccdc80	Coiled-coil domain-containing protein 80	0	rno-miR-141;rno-miR-200a;
26	regulation of chemotaxis	4.21	13	3.09	4.92E-04
		Robo2	Roundabout guidance receptor 2	1	rno-miR-200c;
		Lpar1	Lysophosphatidic acid receptor 1	1	rno-miR-200c;
		Slit2	Slit homolog 2 protein (Fragment)	1	rno-miR-200c;
		Mospd2	Motile sperm domain-containing 2	1	rno-miR-200c;
		Vegfa	Vascular endothelial growth factor A	1	rno-miR-200c;
		Sema5a	Semaphorin-5A	-1	rno-miR-293;
		Il23a	Interleukin-23 subunit alpha	-1	rno-miR-293;
		Dusp1	Dual specificity protein phosphatase 1	1	rno-miR-200c;
		Fn1	Fibronectin	1	rno-miR-200c;
		Pdgfra	Platelet-derived growth factor receptor alpha	0	rno-miR-141;rno-miR-200a;
		Ano6	Anoctamin	0	rno-miR-141;rno-miR-200a;
		Dusp3	Dual-specificity phosphatase 3	0	rno-miR-141;rno-miR-200a;
		Tiam1	T-cell lymphoma invasion and metastasis 1	0	rno-miR-141;rno-miR-200a;
27	learning or memory	6.01	18	3.00	6.33E-05
		Neto1	Neuropilin and tolloid-like 1	-1	rno-miR-293;
		Ap1s2	AP complex subunit sigma	1	rno-miR-200c;
		Rcan2	Calcipressin-2	-1	rno-miR-293;
		Slc6a1	Sodium- and chloride-dependent GABA transporter 1	1	rno-miR-200c;
		Uba6	Similar to RIKEN cDNA 5730469D23 (Predicted)	1	rno-miR-200c;
		Cacna1c	Voltage-dependent L-type calcium channel subunit alpha-1C	1	rno-miR-200c;
		Elavl4	ELAV-like protein 4	1	rno-miR-141;
		Nrg1	Pro-neuregulin-1, membrane-bound isoform	1	rno-miR-200c;
		Ppp1r9b	Neurabin-2	1	rno-miR-200c;
		Jun	Transcription factor AP-1	1	rno-miR-200c;
		Amfr	Autocrine motility factor receptor	1	rno-miR-200c;
		Rps6kb1	Ribosomal protein S6 kinase beta-1	1	rno-miR-200c;
		Aff2	AF4/FMR2 family, member 2	0	rno-miR-141;rno-miR-200a;
		Atp8a1	Phospholipid-transporting ATPase	0	rno-miR-141;rno-miR-200a;
		Cpeb3	Cytoplasmic polyadenylation element-binding protein 3	0	rno-miR-141;rno-miR-200a;
		Cnr1	Cannabinoid receptor 1	0	rno-miR-141;rno-miR-200a;
		Nptn	Neuroplastin	0	rno-miR-141;rno-miR-200a;
		Ctnnd2	Catenin delta-2 (Fragment)	0	rno-miR-141;rno-miR-200a;
28	kidney development	6.10	18	2.95	7.69E-05

		Robo2	Roundabout guidance receptor 2	1	rno-miR-200c;
		Slit2	Slit homolog 2 protein (Fragment)	1	rno-miR-200c;
		Lrp4	Low-density lipoprotein receptor-related protein 4	1	rno-miR-200c;
		Vegfa	Vascular endothelial growth factor A	1	rno-miR-200c;
		Nup107	Nuclear pore complex protein Nup107	1	rno-miR-200c;
		Pkd1	Polycystin 1, transient receptor potential channel-interacting	1	rno-miR-200c;
		Dspp	Dentin sialophosphoprotein	1	rno-miR-200c;
		Tfap2a	Transcription factor AP-2-alpha	1	rno-miR-200c;
		Sulf1	Extracellular sulfatase Sulf-1	1	rno-miR-200c;
		Six4	SIX homeobox 4	-1	rno-miR-293;
		Pdgfra	Platelet-derived growth factor receptor alpha	0	rno-miR-141;rno-miR-200a;
		Tshz3	Teashirt homolog 3	0	rno-miR-141;rno-miR-200a;
		Sim1	Single-minded 1 (Predicted)	0	rno-miR-141;rno-miR-200a;
		Vcan	Versican core protein (Fragments)	0	rno-miR-141;rno-miR-200a;
		Hs2st1	Heparan sulfate 2-O-sulfotransferase 1	0	rno-miR-141;rno-miR-200a;
		Tgfb2	Transforming growth factor beta-2	0	rno-miR-141;rno-miR-200a;
		Sulf2	Extracellular sulfatase	0	rno-miR-141;rno-miR-200a;
		Cyp26b1	Cytochrome P450 26B1	0	rno-miR-141;rno-miR-200a;
29	regulation of Wnt signaling pathway	5.46	16	2.93	2.03E-04
		Lrp4	Low-density lipoprotein receptor-related protein 4	1	rno-miR-200c;
		Dixdc1	Dixin	-1	rno-miR-293;
		Csnk1g3	Casein kinase I isoform gamma-3	1	rno-miR-200c;
		Sema5a	Semaphorin-5A	-1	rno-miR-293;
		Sulf1	Extracellular sulfatase Sulf-1	1	rno-miR-200c;
		Nlk	Serine/threonine-protein kinase NLK	-1	rno-miR-208b-3p;
		Reck	RCG54895, isoform CRA_a	1	rno-miR-200c;
		Ppap2b	Phospholipid phosphatase 3	1	rno-miR-200c;
		Mdfic	MyoD family inhibitor domain containing (Predicted), isoform CRA_a	1	rno-miR-200c;
		Ctnnd2	Catenin delta-2 (Fragment)	0	rno-miR-141;rno-miR-200a;
		Tmem237	Transmembrane protein 237	0	rno-miR-141;rno-miR-200a;
		Nkd1	Naked cuticle homolog 1	0	rno-miR-141;rno-miR-200a;
		Tbl1xr1	Transducin (Beta)-like 1X-linked receptor 1 (Predicted)	0	rno-miR-141;rno-miR-200a;
		Sulf2	Extracellular sulfatase	0	rno-miR-141;rno-miR-200a;
		Tiam1	T-cell lymphoma invasion and metastasis 1	0	rno-miR-141;rno-miR-200a;
		Zeb2	Zinc finger E-box-binding homeobox 2	0	rno-miR-200a;rno-miR-200c;
30	skin development	4.44	13	2.93	7.94E-04
		Lrp4	Low-density lipoprotein receptor-related protein 4	1	rno-miR-200c;
		Acvr1b	Activin receptor type-1B	-1	rno-miR-293;
		Pkd1	Polycystin 1, transient receptor potential channel-interacting	1	rno-miR-200c;
		Dspp	Dentin sialophosphoprotein	1	rno-miR-200c;
		Tfap2a	Transcription factor AP-2-alpha	1	rno-miR-200c;
		Scd1	Acyl-CoA desaturase 1	1	rno-miR-200c;
		Errfi1	ERBB receptor feedback inhibitor 1	1	rno-miR-200c;
		Tp63	Tumor protein 63	-1	rno-miR-293;
		Txnip	Thioredoxin-interacting protein	0	rno-miR-141;rno-miR-200a;
		Ptges3	Prostaglandin E synthase 3	0	rno-miR-141;rno-miR-200a;
		Tgfb2	Transforming growth factor beta-2	0	rno-miR-141;rno-miR-200a;
		Cyp26b1	Cytochrome P450 26B1	0	rno-miR-141;rno-miR-200a;
		Epha2	Eph receptor A2	0	rno-miR-141;rno-miR-200a;
31	regulation of ERK1 and ERK2 cascade	6.52	19	2.92	5.63E-05
		Ranbp9	RAN-binding protein 9	1	rno-miR-200c;
		Vegfa	Vascular endothelial growth factor A	1	rno-miR-200c;
		Fbxw7	F-box/WD repeat-containing protein 7	1	rno-miR-200c;
		Gata4	Transcription factor GATA-4	1	rno-miR-200c;
		Errfi1	ERBB receptor feedback inhibitor 1	1	rno-miR-200c;
		Dusp1	Dual specificity protein phosphatase 1	1	rno-miR-200c;
		Rapgef2	Rap guanine nucleotide exchange factor 2	1	rno-miR-200c;
		Jun	Transcription factor AP-1	1	rno-miR-200c;
		Fn1	Fibronectin	1	rno-miR-200c;
		Pdgfra	Platelet-derived growth factor receptor alpha	0	rno-miR-141;rno-miR-200a;
		Calcr	Calcitonin receptor	0	rno-miR-141;rno-miR-200a;
		Dstyk	Dual serine/threonine and tyrosine protein kinase	0	rno-miR-141;rno-miR-200a;
		Htr2c	5-hydroxytryptamine receptor 2C	0	rno-miR-141;rno-miR-200a;
		Nptn	Neuroplastin	0	rno-miR-141;rno-miR-200a;
		Phlpp1	PH domain leucine-rich repeat protein phosphatase 1	0	rno-miR-141;rno-miR-200a;
		Tnfaip8l3	TNF alpha-induced protein 8-like 3	0	rno-miR-141;rno-miR-200a;
		Dusp3	Dual-specificity phosphatase 3	0	rno-miR-141;rno-miR-200a;
		Tiam1	T-cell lymphoma invasion and metastasis 1	0	rno-miR-141;rno-miR-200a;
		Epha2	Eph receptor A2	0	rno-miR-141;rno-miR-200a;
32	regulation of mRNA metabolic process	4.87	14	2.87	6.01E-04
		Khdrbs1	KH domain-containing, RNA-binding, signal transduction-associated protein 1	1	rno-miR-200c;
		Vegfa	Vascular endothelial growth factor A	1	rno-miR-200c;
		Tob1	Protein Tob1	1	rno-miR-200c;
		Srsf1	RCG34610, isoform CRA_c	1	rno-miR-200c;
		Rbfox1	RNA binding protein fox-1 homolog	1	rno-miR-200c;
		Elavl4	ELAV-like protein 4	1	rno-miR-141;
		Srsf3	RCG61099, isoform CRA_c	-1	rno-miR-293;
		Npm1	Nucleophosmin	1	rno-miR-200c;
		Calcr	Calcitonin receptor	0	rno-miR-141;rno-miR-200a;
		Dcp2	Decapping mRNA 2	0	rno-miR-141;rno-miR-200a;
		Cpeb3	Cytoplasmic polyadenylation element-binding protein 3	0	rno-miR-141;rno-miR-200a;
		Khdrbs2	KH domain-containing, RNA-binding, signal transduction-associated protein 2	0	rno-miR-141;rno-miR-200a;
		Ythdf2	YTH N(6)-methyladenosine RNA-binding protein 2	0	rno-miR-141;rno-miR-200a;
		Supt6h	Transcription elongation factor spt6	0	rno-miR-141;rno-miR-200a;
33	gonad development	5.67	16	2.82	3.06E-04
		Robo2	Roundabout guidance receptor 2	1	rno-miR-200c;
		Slit2	Slit homolog 2 protein (Fragment)	1	rno-miR-200c;
		Cited2	Cbp/p300-interacting transactivator, with Glu/Asp-rich carboxy-terminal domain, 2	1	rno-miR-200c;
		Vegfa	Vascular endothelial growth factor A	1	rno-miR-200c;
		Nup107	Nuclear pore complex protein Nup107	1	rno-miR-200c;
		Arid4b	AT-rich interactive domain-containing protein 4B	1	rno-miR-200c;
		Gata4	Transcription factor GATA-4	1	rno-miR-200c;
		Ermp1	Endoplasmic reticulum metallopeptidase 1	1	rno-miR-200c;
		Six4	SIX homeobox 4	-1	rno-miR-293;
		Acsbg1	Long-chain-fatty-acid--CoA ligase ACSBG1	1	rno-miR-200c;
		Znf830	Zinc finger protein 830	-1	rno-miR-293;
		Pdgfra	Platelet-derived growth factor receptor alpha	0	rno-miR-141;rno-miR-200a;
		Cbl	Cbl proto-oncogene	0	rno-miR-141;rno-miR-200a;
		Klhl10	Kelch-like protein 10	0	rno-miR-141;rno-miR-200a;
		Tgfb2	Transforming growth factor beta-2	0	rno-miR-141;rno-miR-200a;
		Pitx2	Pituitary homeobox 2	0	rno-miR-141;rno-miR-200a;
34	skeletal system morphogenesis	5.05	14	2.77	8.34E-04
		Col9a1	Collagen alpha-1(IX) chain (Fragment)	-1	rno-miR-293;
		Cited2	Cbp/p300-interacting transactivator, with Glu/Asp-rich carboxy-terminal domain, 2	1	rno-miR-200c;
		Mycn	N-myc proto-oncogene protein	-1	rno-miR-293;
		Vegfa	Vascular endothelial growth factor A	1	rno-miR-200c;
		Pkd1	Polycystin 1, transient receptor potential channel-interacting	1	rno-miR-200c;
		Dspp	Dentin sialophosphoprotein	1	rno-miR-200c;
		Tfap2a	Transcription factor AP-2-alpha	1	rno-miR-200c;
		Sulf1	Extracellular sulfatase Sulf-1	1	rno-miR-200c;
		Six4	SIX homeobox 4	-1	rno-miR-293;
		Ndst1	Bifunctional heparan sulfate N-deacetylase/N-sulfotransferase 1	1	rno-miR-200c;
		Pdgfra	Platelet-derived growth factor receptor alpha	0	rno-miR-141;rno-miR-200a;
		Tgfb2	Transforming growth factor beta-2	0	rno-miR-141;rno-miR-200a;
		Cyp26b1	Cytochrome P450 26B1	0	rno-miR-141;rno-miR-200a;
		Zeb1	Zinc finger E-box-binding homeobox 1	0	rno-miR-200a;rno-miR-200c;
35	in utero embryonic development	9.06	25	2.76	9.59E-06
		Slit2	Slit homolog 2 protein (Fragment)	1	rno-miR-200c;
		Cited2	Cbp/p300-interacting transactivator, with Glu/Asp-rich carboxy-terminal domain, 2	1	rno-miR-200c;
		Zfpm2	Zinc finger protein, multitype 2	1	rno-miR-200c;
		Prdm1	PR domain containing 1, with ZNF domain (Predicted)	1	rno-miR-200c;
		Vegfa	Vascular endothelial growth factor A	1	rno-miR-200c;
		Vash1	Vasohibin 1	1	rno-miR-200c;
		Acvr1b	Activin receptor type-1B	-1	rno-miR-293;
		Tpm1	Tropomyosin alpha-1 chain	-1	rno-miR-293;
		Pkd1	Polycystin 1, transient receptor potential channel-interacting	1	rno-miR-200c;
		Srsf1	RCG34610, isoform CRA_c	1	rno-miR-200c;
		Gata4	Transcription factor GATA-4	1	rno-miR-200c;
		Tm4sf1	Transmembrane 4 L six family member 1	-1	rno-miR-293;
		Kdm6a	Lysine demethylase 6A	-1	rno-miR-293;
		Znf830	Zinc finger protein 830	-1	rno-miR-293;
		Matr3	Matrin-3	1	rno-miR-200c;
		Amot	Angiomotin	1	rno-miR-141;
		Pdgfra	Platelet-derived growth factor receptor alpha	0	rno-miR-141;rno-miR-200a;
		Tshz3	Teashirt homolog 3	0	rno-miR-141;rno-miR-200a;
		Pitpnb	Phosphatidylinositol transfer protein beta isoform	0	rno-miR-141;rno-miR-200a;

		Myh10	Myosin-10	0	rno-miR-141;rno-miR-200a;
		Tbl1xr1	Transducin (Beta)-like 1X-linked receptor 1 (Predicted)	0	rno-miR-141;rno-miR-200a;
		Grb2	Growth factor receptor-bound protein 2	0	rno-miR-141;rno-miR-200a;
		Pitx2	Pituitary homeobox 2	0	rno-miR-141;rno-miR-200a;
		Dusp3	Dual-specificity phosphatase 3	0	rno-miR-141;rno-miR-200a;
		Supt6h	Transcription elongation factor spt6	0	rno-miR-141;rno-miR-200a;
36	regulation of developmental growth	7.67	21	2.74	5.50E-05
		Slit2	Slit homolog 2 protein (Fragment)	1	rno-miR-200c;
		Cited2	Cbp/p300-interacting transactivator, with Glu/Asp-rich carboxy-terminal domain, 2	1	rno-miR-200c;
		Zfpm2	Zinc finger protein, multitype 2	1	rno-miR-200c;
		Vegfa	Vascular endothelial growth factor A	1	rno-miR-200c;
		Sema5a	Semaphorin-5A	-1	rno-miR-293;
		Dspp	Dentin sialophosphoprotein	1	rno-miR-200c;
		Gata4	Transcription factor GATA-4	1	rno-miR-200c;
		Cacna1c	Voltage-dependent L-type calcium channel subunit alpha-1C	1	rno-miR-200c;
		Sgip1	SH3-containing GRB2-like protein 3-interacting protein 1	1	rno-miR-200c;
		Nrg1	Pro-neuregulin-1, membrane-bound isoform	1	rno-miR-200c;
		Wwc3	WWC family member 3	1	rno-miR-200c;
		Six4	SIX homeobox 4	-1	rno-miR-293;
		Mllt4	Afadin	-1	rno-miR-293;
		Rab21	Ras-related protein Rab-21	1	rno-miR-200c;
		Fn1	Fibronectin	1	rno-miR-200c;
		Nr3c1	Glucocorticoid receptor	1	rno-miR-141;rno-miR-200a;rno-miR-200c;
		Rps6kb1	Ribosomal protein S6 kinase beta-1	1	rno-miR-200c;
		Nkd1	Naked cuticle homolog 1	0	rno-miR-141;rno-miR-200a;
		Siah1a	E3 ubiquitin-protein ligase SIAH1	0	rno-miR-141;rno-miR-200a;
		Ulk2	Unc-51-like autophagy-activating kinase 2	0	rno-miR-141;rno-miR-200a;
		Clasp2	CLIP-associating protein 2	0	rno-miR-141;rno-miR-200a;
37	negative regulation of growth	5.52	15	2.72	6.69E-04
		Sesn1	Sestrin 1	1	rno-miR-200c;
		Slit2	Slit homolog 2 protein (Fragment)	1	rno-miR-200c;
		Cited2	Cbp/p300-interacting transactivator, with Glu/Asp-rich carboxy-terminal domain, 2	1	rno-miR-200c;
		Acvr1b	Activin receptor type-1B	-1	rno-miR-293;
		Sema5a	Semaphorin-5A	-1	rno-miR-293;
		Dspp	Dentin sialophosphoprotein	1	rno-miR-200c;
		Scd1	Acyl-CoA desaturase 1	1	rno-miR-200c;
		Fhl1	Four and a half LIM domains protein 1	1	rno-miR-200c;
		Eaf2	ELL-associated factor 2	-1	rno-miR-293;
		Ppp1r9b	Neurabin-2	1	rno-miR-200c;
		Wwc3	WWC family member 3	1	rno-miR-200c;
		Apbb2	Amyloid beta precursor protein-binding family B member 2	0	rno-miR-141;rno-miR-200a;
		Nkd1	Naked cuticle homolog 1	0	rno-miR-141;rno-miR-200a;
		Tgfb2	Transforming growth factor beta-2	0	rno-miR-141;rno-miR-200a;
		Ulk2	Unc-51-like autophagy-activating kinase 2	0	rno-miR-141;rno-miR-200a;
38	dephosphorylation	5.54	15	2.71	6.92E-04
		Ptpn13	Protein tyrosine phosphatase, non-receptor type 13	1	rno-miR-200c;
		Ppp4r2	Protein phosphatase 4, regulatory subunit 2	1	rno-miR-200c;
		Hdhd2	Haloacid dehalogenase-like hydrolase domain-containing protein 2	1	rno-miR-200c;
		Ppap2b	Phospholipid phosphatase 3	1	rno-miR-200c;
		Dusp1	Dual specificity protein phosphatase 1	1	rno-miR-200c;
		Lpin3	Lipin 3	1	rno-miR-200c;
		Ptpra	Receptor-type tyrosine-protein phosphatase alpha	-1	rno-miR-200a;
		Ptpn21	Tyrosine-protein phosphatase non-receptor type 21	1	rno-miR-200c;
		Ppp1r15b	Protein phosphatase 1, regulatory (Inhibitor) subunit 15b (Predicted)	0	rno-miR-141;rno-miR-200a;
		Ppm1l	Protein phosphatase, Mg2+/Mn2+-dependent, 1L	0	rno-miR-141;rno-miR-200a;
		Sacm1l	Phosphatidylinositide phosphatase SAC1	0	rno-miR-141;rno-miR-200a;
		Ptp4a1	Protein tyrosine phosphatase type IVA 1	0	rno-miR-141;rno-miR-200a;
		Cdc14a	Cell division cycle 14A	0	rno-miR-141;rno-miR-200a;
		Phlpp1	PH domain leucine-rich repeat protein phosphatase 1	0	rno-miR-141;rno-miR-200a;
		Dusp3	Dual-specificity phosphatase 3	0	rno-miR-141;rno-miR-200a;
39	response to ketone	6.79	18	2.65	2.69E-04
		Slit2	Slit homolog 2 protein (Fragment)	1	rno-miR-200c;
		Slco1a1	Solute carrier organic anion transporter family member 1A1	1	rno-miR-200c;
		Rock2	Rho-associated protein kinase 2	1	rno-miR-200c;
		Vegfa	Vascular endothelial growth factor A	1	rno-miR-200c;
		Dspp	Dentin sialophosphoprotein	1	rno-miR-200c;
		Errfi1	ERBB receptor feedback inhibitor 1	1	rno-miR-200c;
		Nrg1	Pro-neuregulin-1, membrane-bound isoform	1	rno-miR-200c;
		Ppp1r9b	Neurabin-2	1	rno-miR-200c;
		Dusp1	Dual specificity protein phosphatase 1	1	rno-miR-200c;
		Fn1	Fibronectin	1	rno-miR-200c;
		Nr3c1	Glucocorticoid receptor	1	rno-miR-141;rno-miR-200a;rno-miR-200c;
		Rps6kb1	Ribosomal protein S6 kinase beta-1	1	rno-miR-200c;
		Adcy2	Adenylate cyclase type 2	1	rno-miR-200c;
		Npm1	Nucleophosmin	1	rno-miR-200c;
		Cbl	Cbl proto-oncogene	0	rno-miR-141;rno-miR-200a;
		Txnip	Thioredoxin-interacting protein	0	rno-miR-141;rno-miR-200a;
		Klf6	Krueppel-like factor 6	0	rno-miR-141;rno-miR-200a;
		Tgfb2	Transforming growth factor beta-2	0	rno-miR-141;rno-miR-200a;
40	epithelial tube morphogenesis	6.95	18	2.59	3.48E-04
		Slit2	Slit homolog 2 protein (Fragment)	1	rno-miR-200c;
		Cited2	Cbp/p300-interacting transactivator, with Glu/Asp-rich carboxy-terminal domain, 2	1	rno-miR-200c;
		Rock2	Rho-associated protein kinase 2	1	rno-miR-200c;
		Mycn	N-myc proto-oncogene protein	-1	rno-miR-293;
		Vegfa	Vascular endothelial growth factor A	1	rno-miR-200c;
		Pkd1	Polycystin 1, transient receptor potential channel-interacting	1	rno-miR-200c;
		Sema5a	Semaphorin-5A	-1	rno-miR-293;
		Dspp	Dentin sialophosphoprotein	1	rno-miR-200c;
		Tfap2a	Transcription factor AP-2-alpha	1	rno-miR-200c;
		Gata4	Transcription factor GATA-4	1	rno-miR-200c;
		Shroom3	Shroom family member 3	-1	rno-miR-293;
		Kdm6a	Lysine demethylase 6A	-1	rno-miR-293;
		Nr3c1	Glucocorticoid receptor	1	rno-miR-141;rno-miR-200a;rno-miR-200c;
		Hs2st1	Heparan sulfate 2-O-sulfotransferase 1	0	rno-miR-141;rno-miR-200a;
		Tgfb2	Transforming growth factor beta-2	0	rno-miR-141;rno-miR-200a;
		Pitx2	Pituitary homeobox 2	0	rno-miR-141;rno-miR-200a;
		Epha2	Eph receptor A2	0	rno-miR-141;rno-miR-200a;
		Zeb2	Zinc finger E-box-binding homeobox 2	0	rno-miR-200a;rno-miR-200c;
41	supramolecular fiber organization	8.49	22	2.59	7.96E-05
		Clasp1	Cytoplasmic linker-associated protein 1	1	rno-miR-200c;
		Tpm1	Tropomyosin alpha-1 chain	-1	rno-miR-293;
		Spast	Spastin	-1	rno-miR-293;
		Eps8	Epidermal growth factor receptor kinase substrate 8	1	rno-miR-200c;
		Hook1	Hook homolog 1 (Drosophila) (Predicted)	1	rno-miR-200c;
		Shroom3	Shroom family member 3	-1	rno-miR-293;
		Ppp1r9b	Neurabin-2	1	rno-miR-200c;
		Six4	SIX homeobox 4	-1	rno-miR-293;
		Marcks	Myristoylated alanine-rich C-kinase substrate	1	rno-miR-200c;
		Agfg1	Arf-GAP domain and FG repeat-containing protein 1	1	rno-miR-200c;
		Dpt	Dermatopontin	-1	rno-miR-293;
		Tchh	Trichohyalin	-1	rno-miR-293;
		Pls3	Plastin-3	1	rno-miR-200c;
		Tmod3	RCG25684, isoform CRA_a	1	rno-miR-200c;
		Pdgfra	Platelet-derived growth factor receptor alpha	0	rno-miR-141;rno-miR-200a;
		Arpc5	Actin-related protein 2/3 complex subunit 5	0	rno-miR-141;rno-miR-200a;
		Cep120	Centrosomal protein 120	0	rno-miR-141;rno-miR-200a;
		Slain2	SLAIN motif family, member 2	0	rno-miR-141;rno-miR-200a;
		Myh10	Myosin-10	0	rno-miR-141;rno-miR-200a;
		Tgfb2	Transforming growth factor beta-2	0	rno-miR-141;rno-miR-200a;
		Chp1	Calcineurin B homologous protein 1	0	rno-miR-141;rno-miR-200a;
		Clasp2	CLIP-associating protein 2	0	rno-miR-141;rno-miR-200a;
42	transmembrane receptor protein tyrosine kinase signaling pathway	6.57	17	2.59	5.16E-04
		Vegfa	Vascular endothelial growth factor A	1	rno-miR-200c;
		Sulf1	Extracellular sulfatase Sulf-1	1	rno-miR-200c;
		Appl1	Adaptor protein, phosphotyrosine-interacting with PH domain and leucine zipper 1	1	rno-miR-200c;
		Chn1	N-chimaerin	1	rno-miR-200c;
		Nrg1	Pro-neuregulin-1, membrane-bound isoform	1	rno-miR-200c;
		Rapgef2	Rap guanine nucleotide exchange factor 2	1	rno-miR-200c;
		Lcp2	Lymphocyte cytosolic protein 2	1	rno-miR-200c;
		Ndst1	Bifunctional heparan sulfate N-deacetylase/N-sulfotransferase 1	1	rno-miR-200c;
		Ptpra	Receptor-type tyrosine-protein phosphatase alpha	-1	rno-miR-200a;
		Pdgfra	Platelet-derived growth factor receptor alpha	0	rno-miR-141;rno-miR-200a;
		Irs2	Insulin receptor substrate 2	0	rno-miR-141;rno-miR-200a;
		Txnip	Thioredoxin-interacting protein	0	rno-miR-141;rno-miR-200a;
		Sulf2	Extracellular sulfatase	0	rno-miR-141;rno-miR-200a;

		Grb2	Growth factor receptor-bound protein 2	0	rno-miR-141;rno-miR-200a;
		Tiam1	T-cell lymphoma invasion and metastasis 1	0	rno-miR-141;rno-miR-200a;
		Clasp2	CLIP-associating protein 2	0	rno-miR-141;rno-miR-200a;
		Epha2	Eph receptor A2	0	rno-miR-141;rno-miR-200a;
43	negative regulation of locomotion	6.57	17	2.59	5.16E-04
		Robo2	Roundabout guidance receptor 2	1	rno-miR-200c;
		Rap2c	RAP2C, member of RAS oncogene family	1	rno-miR-141;rno-miR-200a;rno-miR-200c;
		Slit2	Slit homolog 2 protein (Fragment)	1	rno-miR-200c;
		Cited2	Cbp/p300-interacting transactivator, with Glu/Asp-rich carboxy-terminal domain, 2	1	rno-miR-200c;
		Clasp1	Cytoplasmic linker-associated protein 1	1	rno-miR-200c;
		Vash1	Vasohibin 1	1	rno-miR-200c;
		Tpm1	Tropomyosin alpha-1 chain	-1	rno-miR-293;
		Sema5a	Semaphorin-5A	-1	rno-miR-293;
		Sulf1	Extracellular sulfatase Sulf-1	1	rno-miR-200c;
		Nrg1	Pro-neuregulin-1, membrane-bound isoform	1	rno-miR-200c;
		Reck	RCG54895, isoform CRA_a	1	rno-miR-200c;
		Mllt4	Afadin	-1	rno-miR-293;
		Dusp1	Dual specificity protein phosphatase 1	1	rno-miR-200c;
		Htr2c	5-hydroxytryptamine receptor 2C	0	rno-miR-141;rno-miR-200a;
		Sept7	Septin-7	0	rno-miR-141;rno-miR-200a;
		Dusp3	Dual-specificity phosphatase 3	0	rno-miR-141;rno-miR-200a;
		Clasp2	CLIP-associating protein 2	0	rno-miR-141;rno-miR-200a;
44	positive regulation of cell cycle	7.10	18	2.53	4.48E-04
		Becn1	Beclin-1	-1	rno-miR-293;
		Cited2	Cbp/p300-interacting transactivator, with Glu/Asp-rich carboxy-terminal domain, 2	1	rno-miR-200c;
		Rock2	Rho-associated protein kinase 2	1	rno-miR-200c;
		Pkd1	Polycystin 1, transient receptor potential channel-interacting	1	rno-miR-200c;
		Mdm4	Protein Mdm4	1	rno-miR-200c;
		Spast	Spastin	-1	rno-miR-293;
		Gata4	Transcription factor GATA-4	1	rno-miR-200c;
		Tp63	Tumor protein 63	-1	rno-miR-293;
		Rps6kb1	Ribosomal protein S6 kinase beta-1	1	rno-miR-200c;
		Npm1	Nucleophosmin	1	rno-miR-200c;
		Tmod3	RCG25684, isoform CRA_a	1	rno-miR-200c;
		Ccnyl1	Cyclin Y-like 1	0	rno-miR-200c;rno-miR-293;
		Cep120	Centrosomal protein 120	0	rno-miR-141;rno-miR-200a;
		Ccne2	Cyclin E2	0	rno-miR-141;rno-miR-200a;
		Tgfb2	Transforming growth factor beta-2	0	rno-miR-141;rno-miR-200a;
		Cdc14a	Cell division cycle 14A	0	rno-miR-141;rno-miR-200a;
		Dusp3	Dual-specificity phosphatase 3	0	rno-miR-141;rno-miR-200a;
		Sfpq	Splicing factor proline and glutamine rich	0	rno-miR-141;rno-miR-200a;
45	cellular response to growth factor stimulus	10.31	25	2.42	9.27E-05
		Becn1	Beclin-1	-1	rno-miR-293;
		Mapk9	Mitogen-activated protein kinase 9	1	rno-miR-200c;
		Cited2	Cbp/p300-interacting transactivator, with Glu/Asp-rich carboxy-terminal domain, 2	1	rno-miR-200c;
		Lrp4	Low-density lipoprotein receptor-related protein 4	1	rno-miR-200c;
		Vegfa	Vascular endothelial growth factor A	1	rno-miR-200c;
		Acvr1b	Activin receptor type-1B	-1	rno-miR-293;
		Gata4	Transcription factor GATA-4	1	rno-miR-200c;
		Nlk	Serine/threonine-protein kinase NLK	-1	rno-miR-208b-3p;
		Errfi1	ERBB receptor feedback inhibitor 1	1	rno-miR-200c;
		Elavl4	ELAV-like protein 4	1	rno-miR-141;
		Appl1	Adaptor protein, phosphotyrosine-interacting with PH domain and leucine zipper 1	1	rno-miR-200c;
		Ppp1r9b	Neurabin-2	1	rno-miR-200c;
		Rapgef2	Rap guanine nucleotide exchange factor 2	1	rno-miR-200c;
		Jun	Transcription factor AP-1	1	rno-miR-200c;
		Fn1	Fibronectin	1	rno-miR-200c;
		Ndst1	Bifunctional heparan sulfate N-deacetylase/N-sulfotransferase 1	1	rno-miR-200c;
		Nr3c1	Glucocorticoid receptor	1	rno-miR-141;rno-miR-200a;rno-miR-200c;
		Rps6kb1	Ribosomal protein S6 kinase beta-1	1	rno-miR-200c;
		Pdgfra	Platelet-derived growth factor receptor alpha	0	rno-miR-141;rno-miR-200a;
		Cbl	Cbl proto-oncogene	0	rno-miR-141;rno-miR-200a;
		Dstyk	Dual serine/threonine and tyrosine protein kinase	0	rno-miR-141;rno-miR-200a;
		Tgfb2	Transforming growth factor beta-2	0	rno-miR-141;rno-miR-200a;
		Grb2	Growth factor receptor-bound protein 2	0	rno-miR-141;rno-miR-200a;
		Dusp3	Dual-specificity phosphatase 3	0	rno-miR-141;rno-miR-200a;
		Zeb1	Zinc finger E-box-binding homeobox 1	0	rno-miR-200a;rno-miR-200c;
46	regulation of cell growth	8.32	20	2.41	5.82E-04
		Sesn1	Sestrin 1	1	rno-miR-200c;
		Slit2	Slit homolog 2 protein (Fragment)	1	rno-miR-200c;
		Vegfa	Vascular endothelial growth factor A	1	rno-miR-200c;
		Acvr1b	Activin receptor type-1B	-1	rno-miR-293;
		Sema5a	Semaphorin-5A	-1	rno-miR-293;
		Bap1	Ubiquitin carboxyl-terminal hydrolase BAP1	1	rno-miR-200c;
		Fhl1	Four and a half LIM domains protein 1	1	rno-miR-200c;
		Nrg1	Pro-neuregulin-1, membrane-bound isoform	1	rno-miR-200c;
		Eaf2	ELL-associated factor 2	-1	rno-miR-293;
		Ppp1r9b	Neurabin-2	1	rno-miR-200c;
		Mllt4	Afadin	-1	rno-miR-293;
		Rab21	Ras-related protein Rab-21	1	rno-miR-200c;
		Fn1	Fibronectin	1	rno-miR-200c;
		Nr3c1	Glucocorticoid receptor	1	rno-miR-141;rno-miR-200a;rno-miR-200c;
		Npm1	Nucleophosmin	1	rno-miR-200c;
		Apbb2	Amyloid beta precursor protein-binding family B member 2	0	rno-miR-141;rno-miR-200a;
		Thrb	Prothrombin	0	rno-miR-141;rno-miR-200a;
		Tgfb2	Transforming growth factor beta-2	0	rno-miR-141;rno-miR-200a;
		Ulk2	Unc-51-like autophagy-activating kinase 2	0	rno-miR-141;rno-miR-200a;
		Clasp2	CLIP-associating protein 2	0	rno-miR-141;rno-miR-200a;
47	forebrain development	9.37	22	2.35	3.55E-04
		Robo2	Roundabout guidance receptor 2	1	rno-miR-200c;
		Lpar1	Lysophosphatidic acid receptor 1	1	rno-miR-200c;
		Slit2	Slit homolog 2 protein (Fragment)	1	rno-miR-200c;
		Dixdc1	Dixin	-1	rno-miR-293;
		Sema5a	Semaphorin-5A	-1	rno-miR-293;
		Tfap2a	Transcription factor AP-2-alpha	1	rno-miR-200c;
		Uba6	Similar to RIKEN cDNA 5730469D23 (Predicted)	1	rno-miR-200c;
		Nrg1	Pro-neuregulin-1, membrane-bound isoform	1	rno-miR-200c;
		Ppp1r9b	Neurabin-2	1	rno-miR-200c;
		Mllt4	Afadin	-1	rno-miR-293;
		Rapgef2	Rap guanine nucleotide exchange factor 2	1	rno-miR-200c;
		Ndst1	Bifunctional heparan sulfate N-deacetylase/N-sulfotransferase 1	1	rno-miR-200c;
		Ttbk2	Tau tubulin kinase 2	0	rno-miR-141;rno-miR-200a;
		Ets1	Protein C-ets-1	0	rno-miR-200c;rno-miR-208b-3p;
		Arpc5	Actin-related protein 2/3 complex subunit 5	0	rno-miR-141;rno-miR-200a;
		Cep120	Centrosomal protein 120	0	rno-miR-141;rno-miR-200a;
		Myh10	Myosin-10	0	rno-miR-141;rno-miR-200a;
		Pitx2	Pituitary homeobox 2	0	rno-miR-141;rno-miR-200a;
		Phlpp1	PH domain leucine-rich repeat protein phosphatase 1	0	rno-miR-141;rno-miR-200a;
		Pcdh9	Protocadherin 9	0	rno-miR-141;rno-miR-200a;
		Zeb1	Zinc finger E-box-binding homeobox 1	0	rno-miR-200a;rno-miR-200c;
		Zeb2	Zinc finger E-box-binding homeobox 2	0	rno-miR-200a;rno-miR-200c;
48	positive regulation of kinase activity	10.00	23	2.30	3.19E-04
		Lpar1	Lysophosphatidic acid receptor 1	1	rno-miR-200c;
		Map4k5	Mitogen-activated protein kinase kinase kinase kinase	1	rno-miR-200c;
		Vegfa	Vascular endothelial growth factor A	1	rno-miR-200c;
		Pkd1	Polycystin 1, transient receptor potential channel-interacting	1	rno-miR-200c;
		Fbxw7	F-box/WD repeat-containing protein 7	1	rno-miR-200c;
		Il23a	Interleukin-23 subunit alpha	-1	rno-miR-293;
		Nrg1	Pro-neuregulin-1, membrane-bound isoform	1	rno-miR-200c;
		Marcks	Myristoylated alanine-rich C-kinase substrate	1	rno-miR-200c;
		Rapgef2	Rap guanine nucleotide exchange factor 2	1	rno-miR-200c;
		Mdfic	MyoD family inhibitor domain containing (Predicted), isoform CRA_a	1	rno-miR-200c;
		Mmd2	Monocyte to macrophage differentiation-associated 2	1	rno-miR-200c;
		Lcp2	Lymphocyte cytosolic protein 2	1	rno-miR-200c;
		Npm1	Nucleophosmin	1	rno-miR-200c;
		Mmd	Monocyte to macrophage differentiation factor	1	rno-miR-200c;
		Ccnyl1	Cyclin Y-like 1	0	rno-miR-200c;rno-miR-293;
		Pdgfra	Platelet-derived growth factor receptor alpha	0	rno-miR-141;rno-miR-200a;
		Dstyk	Dual serine/threonine and tyrosine protein kinase	0	rno-miR-141;rno-miR-200a;
		Thrb	Prothrombin	0	rno-miR-141;rno-miR-200a;
		Cacul1	CDK2-associated and cullin domain-containing protein 1	0	rno-miR-141;rno-miR-200a;
		Tgfb2	Transforming growth factor beta-2	0	rno-miR-141;rno-miR-200a;
		Tnfaip8l3	TNF alpha-induced protein 8-like 3	0	rno-miR-141;rno-miR-200a;
		Tiam1	T-cell lymphoma invasion and metastasis 1	0	rno-miR-141;rno-miR-200a;
		Zeb2	Zinc finger E-box-binding homeobox 2	0	rno-miR-200a;rno-miR-200c;
49	response to wounding	9.45	21	2.22	8.57E-04

		Serpind1	Heparin cofactor 2	-1	rno-miR-293;
		Vegfa	Vascular endothelial growth factor A	1	rno-miR-200c;
		Vash1	Vasohibin 1	1	rno-miR-200c;
		Tpm1	Tropomyosin alpha-1 chain	-1	rno-miR-293;
		Gata4	Transcription factor GATA-4	1	rno-miR-200c;
		Igsf10	Immunoglobulin superfamily member 10	1	rno-miR-200c;
		Nrg1	Pro-neuregulin-1, membrane-bound isoform	1	rno-miR-200c;
		Jun	Transcription factor AP-1	1	rno-miR-200c;
		Fn1	Fibronectin	1	rno-miR-200c;
		Rps6kb1	Ribosomal protein S6 kinase beta-1	1	rno-miR-200c;
		Pdgfra	Platelet-derived growth factor receptor alpha	0	rno-miR-141;rno-miR-200a;
		Thrb	Prothrombin	0	rno-miR-141;rno-miR-200a;
		Ets1	Protein C-ets-1	0	rno-miR-200c;rno-miR-208b-3p;
		Ano6	Anoctamin	0	rno-miR-141;rno-miR-200a;
		Stxbp1	Syntaxin-binding protein 1	0	rno-miR-141;rno-miR-200a;
		Klf6	Krueppel-like factor 6	0	rno-miR-141;rno-miR-200a;
		Vcan	Versican core protein (Fragments)	0	rno-miR-141;rno-miR-200a;
		Serpinb2	Plasminogen activator inhibitor 2 type A	0	rno-miR-141;rno-miR-200a;
		Myh10	Myosin-10	0	rno-miR-141;rno-miR-200a;
		Tgfb2	Transforming growth factor beta-2	0	rno-miR-141;rno-miR-200a;
		Sulf2	Extracellular sulfatase	0	rno-miR-141;rno-miR-200a;
50	regulation of cellular protein localization	10.41	23	2.21	7.23E-04
		Rock2	Rho-associated protein kinase 2	1	rno-miR-200c;
		Lrp4	Low-density lipoprotein receptor-related protein 4	1	rno-miR-200c;
		Vegfa	Vascular endothelial growth factor A	1	rno-miR-200c;
		Rnf168	E3 ubiquitin-protein ligase RNF168	1	rno-miR-200c;
		Fbxw7	F-box/WD repeat-containing protein 7	1	rno-miR-200c;
		Pkia	cAMP-dependent protein kinase inhibitor alpha	1	rno-miR-200c;
		Appl1	Adaptor protein, phosphotyrosine-interacting with PH domain and leucine zipper 1	1	rno-miR-200c;
		Nrg1	Pro-neuregulin-1, membrane-bound isoform	1	rno-miR-200c;
		Ppp1r9b	Neurabin-2	1	rno-miR-200c;
		Mdfic	MyoD family inhibitor domain containing (Predicted), isoform CRA_a	1	rno-miR-200c;
		Npm1	Nucleophosmin	1	rno-miR-200c;
		Ap2b1	AP-2 complex subunit beta	0	rno-miR-141;rno-miR-200a;
		Thrb	Prothrombin	0	rno-miR-141;rno-miR-200a;
		Ttbk2	Tau tubulin kinase 2	0	rno-miR-141;rno-miR-200a;
		Edem1	alpha-1,2-Mannosidase	0	rno-miR-141;rno-miR-200a;
		Nptn	Neuroplastin	0	rno-miR-141;rno-miR-200a;
		Ccne2	Cyclin E2	0	rno-miR-141;rno-miR-200a;
		Lypla1	Acyl-protein thioesterase 1	0	rno-miR-141;rno-miR-200a;
		Tgfb2	Transforming growth factor beta-2	0	rno-miR-141;rno-miR-200a;
		Chp1	Calcineurin B homologous protein 1	0	rno-miR-141;rno-miR-200a;
		Supt6h	Transcription elongation factor spt6	0	rno-miR-141;rno-miR-200a;
		Vamp4	Vesicle-associated membrane protein 4	0	rno-miR-141;rno-miR-200a;
		Epha2	Eph receptor A2	0	rno-miR-141;rno-miR-200a;
51	modulation of chemical synaptic transmission	10.47	23	2.20	7.49E-04
		Neto1	Neuropilin and tolloid-like 1	-1	rno-miR-293;
		Rnf216	Ring finger protein 216	-1	rno-miR-293;
		Slc6a1	Sodium- and chloride-dependent GABA transporter 1	1	rno-miR-200c;
		Git2	G protein-coupled receptor kinase interacting ArfGAP 2	1	rno-miR-200c;
		Elavl4	ELAV-like protein 4	1	rno-miR-141;
		Ppp1r9b	Neurabin-2	1	rno-miR-200c;
		Mllt4	Afadin	-1	rno-miR-293;
		Plcl1	Inactive phospholipase C-like protein 1	1	rno-miR-200c;
		Nptx1	Neuronal pentraxin-1	1	rno-miR-200c;
		Rapgef2	Rap guanine nucleotide exchange factor 2	1	rno-miR-200c;
		Cdh11	Cadherin 11	1	rno-miR-200c;
		Nr3c1	Glucocorticoid receptor	1	rno-miR-141;rno-miR-200a;rno-miR-200c;
		Ptpra	Receptor-type tyrosine-protein phosphatase alpha	-1	rno-miR-200a;
		Tshz3	Teashirt homolog 3	0	rno-miR-141;rno-miR-200a;
		Htr2c	5-hydroxytryptamine receptor 2C	0	rno-miR-141;rno-miR-200a;
		Cpeb3	Cytoplasmic polyadenylation element-binding protein 3	0	rno-miR-141;rno-miR-200a;
		Cnr1	Cannabinoid receptor 1	0	rno-miR-141;rno-miR-200a;
		Pxk	PX domain-containing protein kinase-like protein	0	rno-miR-141;rno-miR-200a;
		Nptn	Neuroplastin	0	rno-miR-141;rno-miR-200a;
		Stxbp1	Syntaxin-binding protein 1	0	rno-miR-141;rno-miR-200a;
		Ctnnd2	Catenin delta-2 (Fragment)	0	rno-miR-141;rno-miR-200a;
		Ywhag	14-3-3 protein gamma	0	rno-miR-141;rno-miR-200a;
		Slc6a9	Sodium- and chloride-dependent glycine transporter 1	0	rno-miR-141;rno-miR-200a;
52	negative regulation of transcription by RNA polymerase II	16.48	36	2.19	2.22E-05
		LOC102556967	Zinc finger protein 484-like	-1	rno-miR-293;
		Cited2	Cbp/p300-interacting transactivator, with Glu/Asp-rich carboxy-terminal domain, 2	1	rno-miR-200c;
		Zfpm2	Zinc finger protein, multitype 2	1	rno-miR-200c;
		Prdm1	PR domain containing 1, with ZNF domain (Predicted)	1	rno-miR-200c;
		Ube2i	SUMO-conjugating enzyme UBC9	1	rno-miR-200c;
		Vegfa	Vascular endothelial growth factor A	1	rno-miR-200c;
		Jazf1	JAZF zinc finger 1	1	rno-miR-141;rno-miR-200a;rno-miR-200c;
		Rnf168	E3 ubiquitin-protein ligase RNF168	1	rno-miR-200c;
		Mdm4	Protein Mdm4	1	rno-miR-200c;
		Pkia	cAMP-dependent protein kinase inhibitor alpha	1	rno-miR-200c;
		Tfap2a	Transcription factor AP-2-alpha	1	rno-miR-200c;
		Hmbox1	Homeobox-containing 1	1	rno-miR-200c;
		Tp63	Tumor protein 63	-1	rno-miR-293;
		Wwc3	WWC family member 3	1	rno-miR-200c;
		Mier3	MIER family member 3	0	rno-miR-200c;rno-miR-208b-3p;
		Dmbx1	Diencephalon/mesencephalon homeobox 1	0	rno-miR-141;rno-miR-200a;
		Tcerg1	Transcription elongation regulator 1	0	rno-miR-141;rno-miR-200a;
		Txnip	Thioredoxin-interacting protein	0	rno-miR-141;rno-miR-200a;
		Hmg20a	High mobility group 20A	0	rno-miR-141;rno-miR-200a;
		Cpeb3	Cytoplasmic polyadenylation element-binding protein 3	0	rno-miR-141;rno-miR-200a;
		Thrb	Thyroid hormone receptor beta	0	rno-miR-141;rno-miR-200a;
		Myt1l	Myelin transcription factor 1-like protein	0	rno-miR-141;rno-miR-200a;
		Dr1	Protein Dr1	0	rno-miR-141;rno-miR-200a;
		Sim2	Single-minded 2 (Predicted)	0	rno-miR-141;rno-miR-200a;
		Ikzf5	IKAROS family zinc finger 5	0	rno-miR-141;rno-miR-200a;
		Tbl1xr1	Transducin (Beta)-like 1X-linked receptor 1 (Predicted)	0	rno-miR-141;rno-miR-200a;
		Sap30l	SAP30-like	0	rno-miR-141;rno-miR-200a;
		Klf12	Kruppel-like factor 12	0	rno-miR-141;rno-miR-200a;
		Crebrf	CREB3 regulatory factor	0	rno-miR-141;rno-miR-200a;
		Pitx2	Pituitary homeobox 2	0	rno-miR-141;rno-miR-200a;
53	protein phosphorylation	15.28	32	2.09	1.25E-04
		Mapk9	Mitogen-activated protein kinase 9	1	rno-miR-200c;
		Tbk1	Similar to TANK-binding kinase 1	1	rno-miR-200c;
		Map4k5	Mitogen-activated protein kinase kinase kinase kinase	1	rno-miR-200c;
		Rock2	Rho-associated protein kinase 2	1	rno-miR-200c;
		Rps6ka2	Ribosomal protein S6 kinase	-1	rno-miR-293;
		Pdik1l	PDLIM1 interacting kinase 1 like (Predicted)	1	rno-miR-200c;
		Csnk1g3	Casein kinase I isoform gamma-3	1	rno-miR-200c;
		Acvr1b	Activin receptor type-1B	-1	rno-miR-293;
		Cdk17	Cyclin-dependent kinase 17	1	rno-miR-141;rno-miR-200a;rno-miR-200c;
		Pkd1	Polycystin 1, transient receptor potential channel-interacting	1	rno-miR-200c;
		Prpf4b	Serine/threonine-protein kinase PRP4 homolog	-1	rno-miR-293;
		Hipk1	Homeodomain-interacting protein kinase 1	1	rno-miR-141;rno-miR-200a;rno-miR-200c;
		Hspa9	Stress-70 protein, mitochondrial	1	rno-miR-200c;
		Nlk	Serine/threonine-protein kinase NLK	-1	rno-miR-208b-3p;
		Nrg1	Pro-neuregulin-1, membrane-bound isoform	1	rno-miR-200c;
		Ppp1r9b	Neurabin-2	1	rno-miR-200c;
		Mdfic	MyoD family inhibitor domain containing (Predicted), isoform CRA_a	1	rno-miR-200c;
		Mmd2	Monocyte to macrophage differentiation-associated 2	1	rno-miR-200c;
		Rps6kb1	Ribosomal protein S6 kinase beta-1	1	rno-miR-200c;
		Pdgfra	Platelet-derived growth factor receptor alpha	0	rno-miR-141;rno-miR-200a;
		Dstyk	Dual serine/threonine and tyrosine protein kinase	0	rno-miR-141;rno-miR-200a;
		Ttbk2	Tau tubulin kinase 2	0	rno-miR-141;rno-miR-200a;
		Pxk	PX domain-containing protein kinase-like protein	0	rno-miR-141;rno-miR-200a;
		Ppm1l	Protein phosphatase, Mg2+/Mn2+-dependent, 1L	0	rno-miR-141;rno-miR-200a;
		Ccne2	Cyclin E2	0	rno-miR-141;rno-miR-200a;
		Tgfb2	Transforming growth factor beta-2	0	rno-miR-141;rno-miR-200a;
		Cdk19	Cell division cycle 2-like 6 (CDK8-like) (Predicted), isoform CRA_c	0	rno-miR-141;rno-miR-200a;
		Ulk2	Unc-51-like autophagy-activating kinase 2	0	rno-miR-141;rno-miR-200a;
		Cdk13	Cyclin-dependent kinase 13	0	rno-miR-141;rno-miR-200a;
		Epha2	Eph receptor A2	0	rno-miR-141;rno-miR-200a;
54	regulation of cellular response to stress	12.91	27	2.09	5.29E-04
		Mapk9	Mitogen-activated protein kinase 9	1	rno-miR-200c;
		Rock2	Rho-associated protein kinase 2	1	rno-miR-200c;
		Vegfa	Vascular endothelial growth factor A	1	rno-miR-200c;
		Dixdc1	Dixin	-1	rno-miR-293;

		Vash1	Vasohibin 1	1	rno-miR-200c;
		Rnf168	E3 ubiquitin-protein ligase RNF168	1	rno-miR-200c;
		Fbxw7	F-box/WD repeat-containing protein 7	1	rno-miR-200c;
		Ppp4r2	Protein phosphatase 4, regulatory subunit 2	1	rno-miR-200c;
		Gata4	Transcription factor GATA-4	1	rno-miR-200c;
		Nrg1	Pro-neuregulin-1, membrane-bound isoform	1	rno-miR-200c;
		Tp63	Tumor protein 63	-1	rno-miR-293;
		Rnf169	Ring finger protein 169	1	rno-miR-200c;
		Dusp1	Dual specificity protein phosphatase 1	1	rno-miR-200c;
		Mdfic	MyoD family inhibitor domain containing (Predicted), isoform CRA_a	1	rno-miR-200c;
		Hyou1	Hypoxia up-regulated protein 1	-1	rno-miR-293;
		Bag5	BAG family molecular chaperone regulator 5	1	rno-miR-200c;
		Npm1	Nucleophosmin	1	rno-miR-200c;
		Ppp1r15b	Protein phosphatase 1, regulatory (Inhibitor) subunit 15b (Predicted)	0	rno-miR-141;rno-miR-200a;
		Edem1	alpha-1,2-Mannosidase	0	rno-miR-141;rno-miR-200a;
		Dek	Protein DEK	0	rno-miR-141;rno-miR-200a;
		Tgfb2	Transforming growth factor beta-2	0	rno-miR-141;rno-miR-200a;
		Crebrf	CREB3 regulatory factor	0	rno-miR-141;rno-miR-200a;
		Phlpp1	PH domain leucine-rich repeat protein phosphatase 1	0	rno-miR-141;rno-miR-200a;
		Dusp3	Dual-specificity phosphatase 3	0	rno-miR-141;rno-miR-200a;
		Tiam1	T-cell lymphoma invasion and metastasis 1	0	rno-miR-141;rno-miR-200a;
		Sfpq	Splicing factor proline and glutamine rich	0	rno-miR-141;rno-miR-200a;
		Zeb2	Zinc finger E-box-binding homeobox 2	0	rno-miR-200a;rno-miR-200c;
55	positive regulation of transcription by RNA polymerase II	24.40	48	1.97	1.36E-05
		LOC102556967	Zinc finger protein 484-like	-1	rno-miR-293;
		Hmgb3	High mobility group box 3	1	rno-miR-200c;
		Tbk1	Similar to TANK-binding kinase 1	1	rno-miR-200c;
		Cited2	Cbp/p300-interacting transactivator, with Glu/Asp-rich carboxy-terminal domain, 2	1	rno-miR-200c;
		Zfpm2	Zinc finger protein, multitype 2	1	rno-miR-200c;
		Mycn	N-myc proto-oncogene protein	-1	rno-miR-293;
		Vegfa	Vascular endothelial growth factor A	1	rno-miR-200c;
		Acvr1b	Activin receptor type-1B	-1	rno-miR-293;
		Tceb1	Elongin-C	1	rno-miR-200c;
		Pkd1	Polycystin 1, transient receptor potential channel-interacting	1	rno-miR-200c;
		Tfap2a	Transcription factor AP-2-alpha	1	rno-miR-200c;
		Il23a	Interleukin-23 subunit alpha	-1	rno-miR-293;
		Arid4b	AT-rich interactive domain-containing protein 4B	1	rno-miR-200c;
		Gata4	Transcription factor GATA-4	1	rno-miR-200c;
		Nrg1	Pro-neuregulin-1, membrane-bound isoform	1	rno-miR-200c;
		Eaf2	ELL-associated factor 2	-1	rno-miR-293;
		Tp63	Tumor protein 63	-1	rno-miR-293;
		Six4	SIX homeobox 4	-1	rno-miR-293;
		Zfp384	Zinc finger protein 384	-1	rno-miR-200a;
		Stag1	Stromal antigen 1	0	rno-miR-141;rno-miR-200a;
		Ets1	Protein C-ets-1	0	rno-miR-200c;rno-miR-208b-3p;
		Thrb	Thyroid hormone receptor beta	0	rno-miR-141;rno-miR-200a;
		Myt1l	Myelin transcription factor 1-like protein	0	rno-miR-141;rno-miR-200a;
		Dr1	Protein Dr1	0	rno-miR-141;rno-miR-200a;
		Ikzf5	IKAROS family zinc finger 5	0	rno-miR-141;rno-miR-200a;
		Klf6	Krueppel-like factor 6	0	rno-miR-141;rno-miR-200a;
		Atxn7	Ataxin 7	0	rno-miR-141;rno-miR-200a;
		Tbl1xr1	Transducin (Beta)-like 1X-linked receptor 1 (Predicted)	0	rno-miR-141;rno-miR-200a;
		Mybl1	MYB proto-oncogene-like 1	0	rno-miR-141;rno-miR-200a;
		Tgfb2	Transforming growth factor beta-2	0	rno-miR-141;rno-miR-200a;
56	positive regulation of protein phosphorylation	19.74	38	1.92	1.79E-04
		Rap2c	RAP2C, member of RAS oncogene family	1	rno-miR-141;rno-miR-200a;rno-miR-200c;
		Lpar1	Lysophosphatidic acid receptor 1	1	rno-miR-200c;
		Mapk9	Mitogen-activated protein kinase 9	1	rno-miR-200c;
		Map4k5	Mitogen-activated protein kinase kinase kinase kinase	1	rno-miR-200c;
		Rock2	Rho-associated protein kinase 2	1	rno-miR-200c;
		Lrp4	Low-density lipoprotein receptor-related protein 4	1	rno-miR-200c;
		Vegfa	Vascular endothelial growth factor A	1	rno-miR-200c;
		Dixdc1	Dixin	-1	rno-miR-293;
		Acvr1b	Activin receptor type-1B	-1	rno-miR-293;
		Pkd1	Polycystin 1, transient receptor potential channel-interacting	1	rno-miR-200c;
		Fbxw7	F-box/WD repeat-containing protein 7	1	rno-miR-200c;
		Rqcd1	CCR4-NOT transcription complex subunit 9	1	rno-miR-200c;
		Il23a	Interleukin-23 subunit alpha	-1	rno-miR-293;
		Gata4	Transcription factor GATA-4	1	rno-miR-200c;
		Nrg1	Pro-neuregulin-1, membrane-bound isoform	1	rno-miR-200c;
		Ppap2b	Phospholipid phosphatase 3	1	rno-miR-200c;
		Marcks	Myristoylated alanine-rich C-kinase substrate	1	rno-miR-200c;
		Rapgef2	Rap guanine nucleotide exchange factor 2	1	rno-miR-200c;
		Mdfic	MyoD family inhibitor domain containing (Predicted), isoform CRA_a	1	rno-miR-200c;
		Jun	Transcription factor AP-1	1	rno-miR-200c;
		Ccnyl1	Cyclin Y-like 1	0	rno-miR-200c;rno-miR-293;
		Pdgfra	Platelet-derived growth factor receptor alpha	0	rno-miR-141;rno-miR-200a;
		Calcr	Calcitonin receptor	0	rno-miR-141;rno-miR-200a;
		Dstyk	Dual serine/threonine and tyrosine protein kinase	0	rno-miR-141;rno-miR-200a;
		Thrb	Prothrombin	0	rno-miR-141;rno-miR-200a;
		Htr2c	5-hydroxytryptamine receptor 2C	0	rno-miR-141;rno-miR-200a;
		Nptn	Neuroplastin	0	rno-miR-141;rno-miR-200a;
		Cacul1	CDK2-associated and cullin domain-containing protein 1	0	rno-miR-141;rno-miR-200a;
		Tgfb2	Transforming growth factor beta-2	0	rno-miR-141;rno-miR-200a;
		Tnfaip8l3	TNF alpha-induced protein 8-like 3	0	rno-miR-141;rno-miR-200a;
57	cellular response to endogenous stimulus	24.07	45	1.87	8.17E-05
		Robo2	Roundabout guidance receptor 2	1	rno-miR-200c;
		Sesn1	Sestrin 1	1	rno-miR-200c;
		Becn1	Beclin-1	-1	rno-miR-293;
		Mapk9	Mitogen-activated protein kinase 9	1	rno-miR-200c;
		Slit2	Slit homolog 2 protein (Fragment)	1	rno-miR-200c;
		Cited2	Cbp/p300-interacting transactivator, with Glu/Asp-rich carboxy-terminal domain, 2	1	rno-miR-200c;
		Rock2	Rho-associated protein kinase 2	1	rno-miR-200c;
		Ugt1a6	UDP-glucuronosyltransferase 1-6	-1	rno-miR-293;
		Lrp4	Low-density lipoprotein receptor-related protein 4	1	rno-miR-200c;
		Vegfa	Vascular endothelial growth factor A	1	rno-miR-200c;
		Tmem38b	Trimeric intracellular cation channel type B	1	rno-miR-200c;
		Acvr1b	Activin receptor type-1B	-1	rno-miR-293;
		Dspp	Dentin sialophosphoprotein	1	rno-miR-200c;
		Gata4	Transcription factor GATA-4	1	rno-miR-200c;
		Nlk	Serine/threonine-protein kinase NLK	-1	rno-miR-208b-3p;
		Cacna1c	Voltage-dependent L-type calcium channel subunit alpha-1C	1	rno-miR-200c;
		Errfi1	ERBB receptor feedback inhibitor 1	1	rno-miR-200c;
		Elavl4	ELAV-like protein 4	1	rno-miR-141;
		Appl1	Adaptor protein, phosphotyrosine-interacting with PH domain and leucine zipper 1	1	rno-miR-200c;
		Olr59	Olfactory receptor 51E2	1	rno-miR-200c;
		Pdgfra	Platelet-derived growth factor receptor alpha	0	rno-miR-141;rno-miR-200a;
		Cbl	Cbl proto-oncogene	0	rno-miR-141;rno-miR-200a;
		Dstyk	Dual serine/threonine and tyrosine protein kinase	0	rno-miR-141;rno-miR-200a;
		Irs2	Insulin receptor substrate 2	0	rno-miR-141;rno-miR-200a;
		Cpeb3	Cytoplasmic polyadenylation element-binding protein 3	0	rno-miR-141;rno-miR-200a;
		Thrb	Thyroid hormone receptor beta	0	rno-miR-141;rno-miR-200a;
		Ptges3	Prostaglandin E synthase 3	0	rno-miR-141;rno-miR-200a;
		Ywhag	14-3-3 protein gamma	0	rno-miR-141;rno-miR-200a;
		Klf6	Krueppel-like factor 6	0	rno-miR-141;rno-miR-200a;
		Tgfb2	Transforming growth factor beta-2	0	rno-miR-141;rno-miR-200a;
58	cytoskeleton organization	19.49	36	1.85	6.34E-04
		Ugt8	2-hydroxyacylsphingosine 1-beta-galactosyltransferase	1	rno-miR-200c;
		Rock2	Rho-associated protein kinase 2	1	rno-miR-200c;
		Rgl1	Lethal(2) giant larvae protein homolog 1	1	rno-miR-200c;
		Clasp1	Cytoplasmic linker-associated protein 1	1	rno-miR-200c;
		Pard6b	Par-6 (Partitioning defective 6) homolog beta (C. elegans) (Predicted)	1	rno-miR-200c;
		Tpm1	Tropomyosin alpha-1 chain	-1	rno-miR-293;
		Spast	Spastin	-1	rno-miR-293;
		Eps8	Epidermal growth factor receptor kinase substrate 8	1	rno-miR-200c;
		Hook1	Hook homolog 1 (Drosophila) (Predicted)	1	rno-miR-200c;
		Shroom3	Shroom family member 3	-1	rno-miR-293;
		Ppp1r9b	Neurabin-2	1	rno-miR-200c;
		Six4	SIX homeobox 4	-1	rno-miR-293;
		Marcks	Myristoylated alanine-rich C-kinase substrate	1	rno-miR-200c;
		Agfg1	Arf-GAP domain and FG repeat-containing protein 1	1	rno-miR-200c;
		Haus1	HAUS augmin-like complex subunit 1	-1	rno-miR-293;
		Tchh	Trichohyalin	-1	rno-miR-293;
		Pls3	Plastin-3	1	rno-miR-200c;
		Pdgfra	Platelet-derived growth factor receptor alpha	0	rno-miR-141;rno-miR-200a;
		Elmo1	Engulfment and cell motility 1	0	rno-miR-141;rno-miR-200a;

		Ttbk2	Tau tubulin kinase 2	0	rno-miR-141;rno-miR-200a;
		Arpc5	Actin-related protein 2/3 complex subunit 5	0	rno-miR-141;rno-miR-200a;
		Map7d1	MAP7 domain-containing 1	0	rno-miR-141;rno-miR-200a;
		Cep120	Centrosomal protein 120	0	rno-miR-141;rno-miR-200a;
		Slain2	SLAIN motif family, member 2	0	rno-miR-141;rno-miR-200a;
		Syne3	Spectrin repeat-containing, nuclear envelope family member 3	0	rno-miR-141;rno-miR-200a;
		Myh10	Myosin-10	0	rno-miR-141;rno-miR-200a;
		Atxn7	Ataxin 7	0	rno-miR-141;rno-miR-200a;
		Sept7	Septin-7	0	rno-miR-141;rno-miR-200a;
		Cdc14a	Cell division cycle 14A	0	rno-miR-141;rno-miR-200a;
		Chp1	Calcineurin B homologous protein 1	0	rno-miR-141;rno-miR-200a;
59	positive regulation of signal transduction	30.07	54	1.80	3.93E-05
		Robo2	Roundabout guidance receptor 2	1	rno-miR-200c;
		Neto1	Neuropilin and tolloid-like 1	-1	rno-miR-293;
		Lpar1	Lysophosphatidic acid receptor 1	1	rno-miR-200c;
		Mapk9	Mitogen-activated protein kinase 9	1	rno-miR-200c;
		Khdrbs1	KH domain-containing, RNA-binding, signal transduction-associated protein 1	1	rno-miR-200c;
		Tbk1	Similar to TANK-binding kinase 1	1	rno-miR-200c;
		Map4k5	Mitogen-activated protein kinase kinase kinase kinase	1	rno-miR-200c;
		Cited2	Cbp/p300-interacting transactivator, with Glu/Asp-rich carboxy-terminal domain, 2	1	rno-miR-200c;
		Rock2	Rho-associated protein kinase 2	1	rno-miR-200c;
		Ube2i	SUMO-conjugating enzyme UBC9	1	rno-miR-200c;
		Vegfa	Vascular endothelial growth factor A	1	rno-miR-200c;
		Dixdc1	Dixin	-1	rno-miR-293;
		Csnk1g3	Casein kinase I isoform gamma-3	1	rno-miR-200c;
		Acvr1b	Activin receptor type-1B	-1	rno-miR-293;
		Zfp423	Zinc finger protein 423	1	rno-miR-141;rno-miR-200a;rno-miR-200c;
		Sema5a	Semaphorin-5A	-1	rno-miR-293;
		Fbxw7	F-box/WD repeat-containing protein 7	1	rno-miR-200c;
		Rqcd1	CCR4-NOT transcription complex subunit 9	1	rno-miR-200c;
		Eps8	Epidermal growth factor receptor kinase substrate 8	1	rno-miR-200c;
		Il23a	Interleukin-23 subunit alpha	-1	rno-miR-293;
		Skp2	S-phase kinase-associated protein 2	1	rno-miR-200c;
		Sulf1	Extracellular sulfatase Sulf-1	1	rno-miR-200c;
		Gata4	Transcription factor GATA-4	1	rno-miR-200c;
		Pdgfra	Platelet-derived growth factor receptor alpha	0	rno-miR-141;rno-miR-200a;
		Calcr	Calcitonin receptor	0	rno-miR-141;rno-miR-200a;
		Cbl	Cbl proto-oncogene	0	rno-miR-141;rno-miR-200a;
		Dstyk	Dual serine/threonine and tyrosine protein kinase	0	rno-miR-141;rno-miR-200a;
		Thrb	Prothrombin	0	rno-miR-141;rno-miR-200a;
		Htr2c	5-hydroxytryptamine receptor 2C	0	rno-miR-141;rno-miR-200a;
		Nptn	Neuroplastin	0	rno-miR-141;rno-miR-200a;
60	response to lipid	23.38	41	1.75	5.95E-04
		Pls3	Phospholipid scramblase 3	1	rno-miR-200c;
		Lpar1	Lysophosphatidic acid receptor 1	1	rno-miR-200c;
		Mapk9	Mitogen-activated protein kinase 9	1	rno-miR-200c;
		Slit2	Slit homolog 2 protein (Fragment)	1	rno-miR-200c;
		Slco1a1	Solute carrier organic anion transporter family member 1A1	1	rno-miR-200c;
		Rock2	Rho-associated protein kinase 2	1	rno-miR-200c;
		Slc6a1	Sodium- and chloride-dependent GABA transporter 1	1	rno-miR-200c;
		Ugt1a6	UDP-glucuronosyltransferase 1-6	-1	rno-miR-293;
		Vegfa	Vascular endothelial growth factor A	1	rno-miR-200c;
		Dspp	Dentin sialophosphoprotein	1	rno-miR-200c;
		Tfap2a	Transcription factor AP-2-alpha	1	rno-miR-200c;
		Scd1	Acyl-CoA desaturase 1	1	rno-miR-200c;
		Gata4	Transcription factor GATA-4	1	rno-miR-200c;
		Errfi1	ERBB receptor feedback inhibitor 1	1	rno-miR-200c;
		Nrg1	Pro-neuregulin-1, membrane-bound isoform	1	rno-miR-200c;
		Olr59	Olfactory receptor 51E2	1	rno-miR-200c;
		Ppp1r9b	Neurabin-2	1	rno-miR-200c;
		Dusp1	Dual specificity protein phosphatase 1	1	rno-miR-200c;
		Pdgfra	Platelet-derived growth factor receptor alpha	0	rno-miR-141;rno-miR-200a;
		Calcr	Calcitonin receptor	0	rno-miR-141;rno-miR-200a;
		Cbl	Cbl proto-oncogene	0	rno-miR-141;rno-miR-200a;
		Txnip	Thioredoxin-interacting protein	0	rno-miR-141;rno-miR-200a;
		Thbd	Thrombomodulin	0	rno-miR-141;rno-miR-200a;
		Ets1	Protein C-ets-1	0	rno-miR-200c;rno-miR-208b-3p;
		Thrb	Thyroid hormone receptor beta	0	rno-miR-141;rno-miR-200a;
		Cnr1	Cannabinoid receptor 1	0	rno-miR-141;rno-miR-200a;
		Stxbp1	Syntaxin-binding protein 1	0	rno-miR-141;rno-miR-200a;
		Ptges3	Prostaglandin E synthase 3	0	rno-miR-141;rno-miR-200a;
		Tgfb2	Transforming growth factor beta-2	0	rno-miR-141;rno-miR-200a;
		Pitx2	Pituitary homeobox 2	0	rno-miR-141;rno-miR-200a;
61	response to hormone	23.54	41	1.74	8.55E-04
		Robo2	Roundabout guidance receptor 2	1	rno-miR-200c;
		Slit2	Slit homolog 2 protein (Fragment)	1	rno-miR-200c;
		Slco1a1	Solute carrier organic anion transporter family member 1A1	1	rno-miR-200c;
		Rock2	Rho-associated protein kinase 2	1	rno-miR-200c;
		Slc6a1	Sodium- and chloride-dependent GABA transporter 1	1	rno-miR-200c;
		Ugt1a6	UDP-glucuronosyltransferase 1-6	-1	rno-miR-293;
		Vegfa	Vascular endothelial growth factor A	1	rno-miR-200c;
		Dspp	Dentin sialophosphoprotein	1	rno-miR-200c;
		Gata4	Transcription factor GATA-4	1	rno-miR-200c;
		Hspa9	Stress-70 protein, mitochondrial	1	rno-miR-200c;
		Errfi1	ERBB receptor feedback inhibitor 1	1	rno-miR-200c;
		Appl1	Adaptor protein, phosphotyrosine-interacting with PH domain and leucine zipper 1	1	rno-miR-200c;
		Nrg1	Pro-neuregulin-1, membrane-bound isoform	1	rno-miR-200c;
		Olr59	Olfactory receptor 51E2	1	rno-miR-200c;
		Ppp1r9b	Neurabin-2	1	rno-miR-200c;
		Dusp1	Dual specificity protein phosphatase 1	1	rno-miR-200c;
		Pdgfra	Platelet-derived growth factor receptor alpha	0	rno-miR-141;rno-miR-200a;
		Calcr	Calcitonin receptor	0	rno-miR-141;rno-miR-200a;
		Cbl	Cbl proto-oncogene	0	rno-miR-141;rno-miR-200a;
		Thrb	Prothrombin	0	rno-miR-141;rno-miR-200a;
		Irs2	Insulin receptor substrate 2	0	rno-miR-141;rno-miR-200a;
		Txnip	Thioredoxin-interacting protein	0	rno-miR-141;rno-miR-200a;
		Ets1	Protein C-ets-1	0	rno-miR-200c;rno-miR-208b-3p;
		Ak2	Adenylate kinase 2, mitochondrial	0	rno-miR-141;rno-miR-200a;
		Thrb	Thyroid hormone receptor beta	0	rno-miR-141;rno-miR-200a;
		Stxbp1	Syntaxin-binding protein 1	0	rno-miR-141;rno-miR-200a;
		Ptges3	Prostaglandin E synthase 3	0	rno-miR-141;rno-miR-200a;
		Ywhag	14-3-3 protein gamma	0	rno-miR-141;rno-miR-200a;
		Tgfb2	Transforming growth factor beta-2	0	rno-miR-141;rno-miR-200a;
		Grb2	Growth factor receptor-bound protein 2	0	rno-miR-141;rno-miR-200a;
62	regulation of cell death	32.93	55	1.67	2.40E-04
		Lpar1	Lysophosphatidic acid receptor 1	1	rno-miR-200c;
		Becn1	Beclin-1	-1	rno-miR-293;
		Mapk9	Mitogen-activated protein kinase 9	1	rno-miR-200c;
		Slit2	Slit homolog 2 protein (Fragment)	1	rno-miR-200c;
		Cited2	Cbp/p300-interacting transactivator, with Glu/Asp-rich carboxy-terminal domain, 2	1	rno-miR-200c;
		Zfpm2	Zinc finger protein, multitype 2	1	rno-miR-200c;
		Rps6ka2	Ribosomal protein S6 kinase	-1	rno-miR-293;
		Mycn	N-myc proto-oncogene protein	-1	rno-miR-293;
		Vegfa	Vascular endothelial growth factor A	1	rno-miR-200c;
		Mdm4	Protein Mdm4	1	rno-miR-200c;
		Sema5a	Semaphorin-5A	-1	rno-miR-293;
		Fbxw7	F-box/WD repeat-containing protein 7	1	rno-miR-200c;
		Dspp	Dentin sialophosphoprotein	1	rno-miR-200c;
		Tfap2a	Transcription factor AP-2-alpha	1	rno-miR-200c;
		Skp2	S-phase kinase-associated protein 2	1	rno-miR-200c;
		Gata4	Transcription factor GATA-4	1	rno-miR-200c;
		Apbb2	Amyloid beta precursor protein-binding family B member 2	0	rno-miR-141;rno-miR-200a;
		Calcr	Calcitonin receptor	0	rno-miR-141;rno-miR-200a;
		Cbl	Cbl proto-oncogene	0	rno-miR-141;rno-miR-200a;
		Dstyk	Dual serine/threonine and tyrosine protein kinase	0	rno-miR-141;rno-miR-200a;
		Unc5c	Netrin receptor UNC5C	0	rno-miR-141;rno-miR-200a;
		Ap2b1	AP-2 complex subunit beta	0	rno-miR-141;rno-miR-200a;
		Irs2	Insulin receptor substrate 2	0	rno-miR-141;rno-miR-200a;
		Txnip	Thioredoxin-interacting protein	0	rno-miR-141;rno-miR-200a;
		Ets1	Protein C-ets-1	0	rno-miR-200c;rno-miR-208b-3p;
		Ano6	Anoctamin	0	rno-miR-141;rno-miR-200a;
		Cnr1	Cannabinoid receptor 1	0	rno-miR-141;rno-miR-200a;
		Stxbp1	Syntaxin-binding protein 1	0	rno-miR-141;rno-miR-200a;
		Ptges3	Prostaglandin E synthase 3	0	rno-miR-141;rno-miR-200a;
		Serpinb2	Plasminogen activator inhibitor 2 type A	0	rno-miR-141;rno-miR-200a;
63	cellular component assembly	43.77	73	1.67	1.84E-05

		Ugt8	2-hydroxyacylsphingosine 1-beta-galactosyltransferase	1	rno-miR-200c;
		Lpar1	Lysophosphatidic acid receptor 1	1	rno-miR-200c;
		Becn1	Beclin-1	-1	rno-miR-293;
		Khdrbs1	KH domain-containing, RNA-binding, signal transduction-associated protein 1	1	rno-miR-200c;
		Coa3	Cytochrome C oxidase assembly factor 3	-1	rno-miR-293;
		Rgl1	Lethal(2) giant larvae protein homolog 1	1	rno-miR-200c;
		Slc6a1	Sodium- and chloride-dependent GABA transporter 1	1	rno-miR-200c;
		Clasp1	Cytoplasmic linker-associated protein 1	1	rno-miR-200c;
		Pard6b	Par-6 (Partitioning defective 6) homolog beta (C. elegans) (Predicted)	1	rno-miR-200c;
		Lrp4	Low-density lipoprotein receptor-related protein 4	1	rno-miR-200c;
		Kcnd2	Potassium voltage-gated channel subfamily D member 2	1	rno-miR-200c;
		Tpm1	Tropomyosin alpha-1 chain	-1	rno-miR-293;
		Nup107	Nuclear pore complex protein Nup107	1	rno-miR-200c;
		Pkd1	Polycystin 1, transient receptor potential channel-interacting	1	rno-miR-200c;
		Mdm4	Protein Mdm4	1	rno-miR-200c;
		Spast	Spastin	-1	rno-miR-293;
		Nap1l5	Nucleosome assembly protein 1-like 5	1	rno-miR-200c;
		Srsf1	RCG34610, isoform CRA_c	1	rno-miR-200c;
		Pdgfra	Platelet-derived growth factor receptor alpha	0	rno-miR-141;rno-miR-200a;
		Nfasc	Neurofascin	0	rno-miR-141;rno-miR-200a;
		Ap2b1	AP-2 complex subunit beta	0	rno-miR-141;rno-miR-200a;
		Ttbk2	Tau tubulin kinase 2	0	rno-miR-141;rno-miR-200a;
		Ano6	Anoctamin	0	rno-miR-141;rno-miR-200a;
		Psmd11	26S proteasome non-ATPase regulatory subunit 11	0	rno-miR-141;rno-miR-200a;
		Oat	Ornithine aminotransferase, mitochondrial	0	rno-miR-200c;rno-miR-293;
		Ikzf5	IKAROS family zinc finger 5	0	rno-miR-141;rno-miR-200a;
		Nptn	Neuroplastin	0	rno-miR-141;rno-miR-200a;
		Stxbp1	Syntaxin-binding protein 1	0	rno-miR-141;rno-miR-200a;
		Ctnnd2	Catenin delta-2 (Fragment)	0	rno-miR-141;rno-miR-200a;
		Ptges3	Prostaglandin E synthase 3	0	rno-miR-141;rno-miR-200a;
64	regulation of cell population proliferation	32.52	52	1.60	9.23E-04
		Becn1	Beclin-1	-1	rno-miR-293;
		Slit2	Slit homolog 2 protein (Fragment)	1	rno-miR-200c;
		Cited2	Cbp/p300-interacting transactivator, with Glu/Asp-rich carboxy-terminal domain, 2	1	rno-miR-200c;
		Zfpm2	Zinc finger protein, multitype 2	1	rno-miR-200c;
		Prdm1	PR domain containing 1, with ZNF domain (Predicted)	1	rno-miR-200c;
		Rps6ka2	Ribosomal protein S6 kinase	-1	rno-miR-293;
		Mycn	N-myc proto-oncogene protein	-1	rno-miR-293;
		Vegfa	Vascular endothelial growth factor A	1	rno-miR-200c;
		Tob1	Protein Tob1	1	rno-miR-200c;
		Vash1	Vasohibin 1	1	rno-miR-200c;
		Tpm1	Tropomyosin alpha-1 chain	-1	rno-miR-293;
		Mdm4	Protein Mdm4	1	rno-miR-200c;
		Sema5a	Semaphorin-5A	-1	rno-miR-293;
		Dspp	Dentin sialophosphoprotein	1	rno-miR-200c;
		Tfap2a	Transcription factor AP-2-alpha	1	rno-miR-200c;
		Il23a	Interleukin-23 subunit alpha	-1	rno-miR-293;
		Skp2	S-phase kinase-associated protein 2	1	rno-miR-200c;
		Hipk1	Homeodomain-interacting protein kinase 1	1	rno-miR-141;rno-miR-200a;rno-miR-200c;
		Sulf1	Extracellular sulfatase Sulf-1	1	rno-miR-200c;
		Gata4	Transcription factor GATA-4	1	rno-miR-200c;
		Adk	Adenosine kinase	1	rno-miR-200c;
		Pdgfra	Platelet-derived growth factor receptor alpha	0	rno-miR-141;rno-miR-200a;
		Thrb	Prothrombin	0	rno-miR-141;rno-miR-200a;
		Irs2	Insulin receptor substrate 2	0	rno-miR-141;rno-miR-200a;
		Txnip	Thioredoxin-interacting protein	0	rno-miR-141;rno-miR-200a;
		Ets1	Protein C-ets-1	0	rno-miR-200c;rno-miR-208b-3p;
		Thrb	Thyroid hormone receptor beta	0	rno-miR-141;rno-miR-200a;
		Pds5b	Sister chromatid cohesion protein PDS5 homolog B	0	rno-miR-141;rno-miR-200a;
		Ccne2	Cyclin E2	0	rno-miR-141;rno-miR-200a;
		Cacul1	CDK2-associated and cullin domain-containing protein 1	0	rno-miR-141;rno-miR-200a;
65	G protein-coupled receptor signaling pathway	40.37	13	0.32	5.04E-07
		Lpar1	Lysophosphatidic acid receptor 1	1	rno-miR-200c;
		Slit2	Slit homolog 2 protein (Fragment)	1	rno-miR-200c;
		Nrg1	Sphingosine 1-phosphate receptor 5	1	rno-miR-200c;
		Olr59	Olfactory receptor 51E2	1	rno-miR-200c;
		Plcl1	Inactive phospholipase C-like protein 1	1	rno-miR-200c;
		Rapgef2	Rap guanine nucleotide exchange factor 2	1	rno-miR-200c;
		Gpr158	Probable G-protein coupled receptor 158	1	rno-miR-200c;
		Adcy2	Adenylate cyclase type 2	1	rno-miR-200c;
		Calcr	Calcitonin receptor	0	rno-miR-141;rno-miR-200a;
		Htr2c	5-hydroxytryptamine receptor 2C	0	rno-miR-141;rno-miR-200a;
		Cnr1	Cannabinoid receptor 1	0	rno-miR-141;rno-miR-200a;
		Atrnl1	Attractin-like 1	0	rno-miR-141;rno-miR-200a;
		Gabra2	Gamma-aminobutyric acid receptor subunit alpha-2	0	rno-miR-141;rno-miR-200a;
66	oxidation-reduction process	17.75	5	0.28	7.42E-04
		Sesn1	Sestrin 1	1	rno-miR-200c;
		RGD1304810	Similar to 6430573F11Rik protein	1	rno-miR-200c;
		Scd1	Acyl-CoA desaturase 1	1	rno-miR-200c;
		Ptges3	Prostaglandin E synthase 3	0	rno-miR-141;rno-miR-200a;
		Cyp26b1	Cytochrome P450 26B1	0	rno-miR-141;rno-miR-200a;
67	detection of chemical stimulus involved in sensory perception of smell	22.70	1	0.04	3.90E-09
		Olr59	Olfactory receptor 51E2	1	rno-miR-200c;
