# Supplementary material for: Cardiac miRNA Expression and their mRNA Targets in a Rat Model of Prediabetes
Source: Int J Mol Sci. 2020 Mar 20;21(6):2128. doi: 10.3390/ijms21062128 (PMC7139428; doi:10.3390/ijms21062128)
Supplement: Supplementary file 1 [file ijms-21-02128-s001.zip › sTable 1 .pdf]

Target_entrez_id	Target_symbol	Degree	Upreg_degree	Downreg_degree	Node_strength	Associated miRNAs
24413	Nr3c1	3	2	1	1	rno-miR-141;rno-miR-200a;rno-miR-200c;
286973	Elavl2	3	2	1	1	rno-miR-141;rno-miR-200a;rno-miR-200c;
301415	Ankrd44	3	2	1	1	rno-miR-141;rno-miR-200a;rno-miR-200c;
302495	Rap2c	3	2	1	1	rno-miR-141;rno-miR-200a;rno-miR-200c;
314743	Cdk17	3	2	1	1	rno-miR-141;rno-miR-200a;rno-miR-200c;
317599	Atp11c	3	2	1	1	rno-miR-141;rno-miR-200a;rno-miR-200c;
360511	Pank3	3	1	2	-1	rno-miR-141;rno-miR-200a;rno-miR-293;
365895	Hipk1	3	2	1	1	rno-miR-141;rno-miR-200a;rno-miR-200c;
498160	Zkscan1	3	1	2	-1	rno-miR-141;rno-miR-200a;rno-miR-293;
685879	Jazf1	3	2	1	1	rno-miR-141;rno-miR-200a;rno-miR-200c;
94188	Zfp423	3	2	1	1	rno-miR-141;rno-miR-200a;rno-miR-200c;
100302372	LOC100302372	2	1	1	0	rno-miR-141;rno-miR-200a;
100909830	LOC100909830	2	1	1	0	rno-miR-141;rno-miR-200a;
100912052	LOC100912052	2	1	1	0	rno-miR-141;rno-miR-200a;
102557163	LOC102557163	2	1	1	0	rno-miR-141;rno-miR-200a;
114028	Ctnnd2	2	1	1	0	rno-miR-141;rno-miR-200a;
114122	Vcan	2	1	1	0	rno-miR-141;rno-miR-200a;
114216	S100a3	2	1	1	0	rno-miR-141;rno-miR-200a;
114514	Clasp2	2	1	1	0	rno-miR-141;rno-miR-200a;
114557	Wif1	2	1	1	0	rno-miR-141;rno-miR-200a;
114561	Pitpnb	2	1	1	0	rno-miR-141;rno-miR-200a;
114861	Scpep1	2	1	1	0	rno-miR-141;rno-miR-200a;
116482	Sacm1l	2	1	1	0	rno-miR-141;rno-miR-200a;
116506	Calcr	2	1	1	0	rno-miR-141;rno-miR-200a;
116509	Slc6a9	2	1	1	0	rno-miR-141;rno-miR-200a;
116668	Myt1l	2	1	1	0	rno-miR-141;rno-miR-200a;
116690	Nfasc	2	1	1	0	rno-miR-141;rno-miR-200a;
117514	Txnip	2	1	1	0	rno-miR-141;rno-miR-200a;
140670	Ap2b1	2	1	1	0	rno-miR-141;rno-miR-200a;
140941	Siah1a	2	1	1	0	rno-miR-141;rno-miR-200a;
170843	Khdrbs2	2	1	1	0	rno-miR-141;rno-miR-200a;
171517	Gpc2	2	1	1	0	rno-miR-141;rno-miR-200a;
24184	Ak2	2	1	1	0	rno-miR-141;rno-miR-200a;
24356	Ets1	2	1	1	0	rno-miR-200c;rno-miR-208b-3p;
24398	Gls	2	1	1	0	rno-miR-141;rno-miR-200a;
24831	Thrb	2	1	1	0	rno-miR-141;rno-miR-200a;
25187	Htr2c	2	1	1	0	rno-miR-141;rno-miR-200a;
25248	Cnr1	2	1	1	0	rno-miR-141;rno-miR-200a;
25267	Pdgfra	2	1	1	0	rno-miR-141;rno-miR-200a;
252855	Sfpq	2	1	1	0	rno-miR-141;rno-miR-200a;
25514	Lypla1	2	1	1	0	rno-miR-141;rno-miR-200a;
25558	Stxbp1	2	1	1	0	rno-miR-141;rno-miR-200a;
25705	Zeb1	2	1	1	0	rno-miR-200a;rno-miR-200c;
287212	Rnf145	2	1	1	0	rno-miR-141;rno-miR-200a;
287275	Sec24a	2	1	1	0	rno-miR-200c;rno-miR-293;
288264	Ifnar1	2	1	1	0	rno-miR-141;rno-miR-200a;
288702	Unc119b	2	1	1	0	rno-miR-141;rno-miR-200a;
289084	RGD1309104	2	1	1	0	rno-miR-141;rno-miR-200a;
289606	Gabra2	2	1	1	0	rno-miR-141;rno-miR-200a;
289615	Atp8a1	2	1	1	0	rno-miR-141;rno-miR-200a;
289633	RGD1563065	2	1	1	0	rno-miR-141;rno-miR-200a;
289881	Dr1	2	1	1	0	rno-miR-141;rno-miR-200a;
289883	Tmed5	2	1	1	0	rno-miR-141;rno-miR-200a;
291320	Fam188a	2	1	1	0	rno-miR-141;rno-miR-200a;
291534	Rnmt	2	1	1	0	rno-miR-141;rno-miR-200a;
291604	Dcp2	2	1	1	0	rno-miR-141;rno-miR-200a;
29170	Aqp6	2	1	1	0	rno-miR-141;rno-miR-200a;
291925	RGD1308706	2	1	1	0	rno-miR-141;rno-miR-200a;
292155	Hs2st1	2	1	1	0	rno-miR-141;rno-miR-200a;
292947	RGD1309903	2	1	1	0	rno-miR-141;rno-miR-200a;
293098	Tmem135	2	1	1	0	rno-miR-141;rno-miR-200a;
29376	Irs2	2	1	1	0	rno-miR-141;rno-miR-200a;
293922	Aff2	2	1	1	0	rno-miR-141;rno-miR-200a;
294307	Kctd20	2	1	1	0	rno-miR-141;rno-miR-200a;
29463	Ptp4a1	2	1	1	0	rno-miR-141;rno-miR-200a;
29735	Slc16a7	2	1	1	0	rno-miR-141;rno-miR-200a;
297504	Edem1	2	1	1	0	rno-miR-141;rno-miR-200a;
297783	Mybl1	2	1	1	0	rno-miR-141;rno-miR-200a;
298247	Mysm1	2	1	1	0	rno-miR-141;rno-miR-200a;
298792	Ypel5	2	1	1	0	rno-miR-141;rno-miR-200a;
299103	Klhl28	2	1	1	0	rno-miR-141;rno-miR-200a;
299356	Syne3	2	1	1	0	rno-miR-141;rno-miR-200a;
300231	Dip2b	2	1	1	0	rno-miR-141;rno-miR-200a;
302288	Fem1c	2	1	1	0	rno-miR-141;rno-miR-200a;
302669	Car5b	2	1	1	0	rno-miR-141;rno-miR-200a;
303016	Crebrf	2	1	1	0	rno-miR-141;rno-miR-200a;
303206	Ulk2	2	1	1	0	rno-miR-141;rno-miR-200a;
303281	Supt6h	2	1	1	0	rno-miR-141;rno-miR-200a;
303353	Psmd11	2	1	1	0	rno-miR-141;rno-miR-200a;
303533	Klhl10	2	1	1	0	rno-miR-141;rno-miR-200a;
304071	Sim2	2	1	1	0	rno-miR-141;rno-miR-200a;
304109	Tiam1	2	1	1	0	rno-miR-141;rno-miR-200a;
304218	Pds5b	2	1	1	0	rno-miR-141;rno-miR-200a;
304791	Dstyk	2	1	1	0	rno-miR-141;rno-miR-200a;
304799	Ppp1r15b	2	1	1	0	rno-miR-141;rno-miR-200a;
304900	Astn1	2	1	1	0	rno-miR-141;rno-miR-200a;

305310	Slain2	2	1	1	0 rno-miR-141;rno-miR-200a;
305338	Apbb2	2	1	1	0 rno-miR-141;rno-miR-200a;
305911	RGD1306353	2	1	1	0 rno-miR-141;rno-miR-200a;
306091	Pcdh9	2	1	1	0 rno-miR-141;rno-miR-200a;
306110	Klf12	2	1	1	0 rno-miR-141;rno-miR-200a;
306203	Pxk	2	1	1	0 rno-miR-141;rno-miR-200a;
306720	Ctsm	2	1	1	0 rno-miR-141;rno-miR-200a;
306817	Dek	2	1	1	0 rno-miR-141;rno-miR-200a;
306998	Cdk13	2	1	1	0 rno-miR-141;rno-miR-200a;
307302	Cep120	2	1	1	0 rno-miR-141;rno-miR-200a;
307474	Tcerg1	2	1	1	0 rno-miR-141;rno-miR-200a;
307992	Atrnl1	2	1	1	0 rno-miR-141;rno-miR-200a;
308523	Tshz3	2	1	1	0 rno-miR-141;rno-miR-200a;
309031	Ikzf5	2	1	1	0 rno-miR-141;rno-miR-200a;
309126	Tnpo1	2	1	1	0 rno-miR-141;rno-miR-200a;
309326	Ranbp6	2	1	1	0 rno-miR-141;rno-miR-200a;
309510	Cpeb3	2	1	1	0 rno-miR-141;rno-miR-200a;
309804	Cdk19	2	1	1	0 rno-miR-141;rno-miR-200a;
309812	Rev3l	2	1	1	0 rno-miR-141;rno-miR-200a;
309888	Sim1	2	1	1	0 rno-miR-141;rno-miR-200a;
310086	Mier3	2	1	1	0 rno-miR-200c;rno-miR-208b-3p;
310506	Ppm1l	2	1	1	0 rno-miR-141;rno-miR-200a;
310806	Cdc14a	2	1	1	0 rno-miR-141;rno-miR-200a;
311071	Zeb2	2	1	1	0 rno-miR-200a;rno-miR-200c;
311349	Ttbk2	2	1	1	0 rno-miR-141;rno-miR-200a;
311415	Zc3h6	2	1	1	0 rno-miR-141;rno-miR-200a;
311642	Sulf2	2	1	1	0 rno-miR-141;rno-miR-200a;
311743	Zak	2	1	1	0 rno-miR-141;rno-miR-200a;
312135	Tmem168	2	1	1	0 rno-miR-141;rno-miR-200a;
312495	Cyp26b1	2	1	1	0 rno-miR-141;rno-miR-200a;
312828	Etnk1	2	1	1	0 rno-miR-141;rno-miR-200a;
312903	Tram1	2	1	1	0 rno-miR-141;rno-miR-200a;
313053	Ythdf2	2	1	1	0 rno-miR-141;rno-miR-200a;
313512	Dmbx1	2	1	1	0 rno-miR-141;rno-miR-200a;
315272	Ano6	2	1	1	0 rno-miR-141;rno-miR-200a;
315327	Spryd3	2	1	1	0 rno-miR-141;rno-miR-200a;
315608	Ube4a	2	1	1	0 rno-miR-141;rno-miR-200a;
315673	Tnfaip8l3	2	1	1	0 rno-miR-141;rno-miR-200a;
315689	Hmg20a	2	1	1	0 rno-miR-141;rno-miR-200a;
315741	Paqr5	2	1	1	0 rno-miR-141;rno-miR-200a;
315958	Stag1	2	1	1	0 rno-miR-141;rno-miR-200a;
315959	Msl2	2	1	1	0 rno-miR-200c;rno-miR-293;
316012	Klhl18	2	1	1	0 rno-miR-141;rno-miR-200a;
316412	Tmem237	2	1	1	0 rno-miR-141;rno-miR-200a;
316452	Ccnyl1	2	1	1	0 rno-miR-200c;rno-miR-293;
316764	Soga2	2	1	1	0 rno-miR-141;rno-miR-200a;
360531	Sap30l	2	1	1	0 rno-miR-141;rno-miR-200a;
360854	Arpc5	2	1	1	0 rno-miR-141;rno-miR-200a;
360937	Tbc1d1	2	1	1	0 rno-miR-141;rno-miR-200a;
361015	Atxn7	2	1	1	0 rno-miR-141;rno-miR-200a;
361016	LOC361016	2	1	1	0 rno-miR-141;rno-miR-200a;
361251	Elmo1	2	1	1	0 rno-miR-141;rno-miR-200a;
361442	Sipa1l2	2	1	1	0 rno-miR-141;rno-miR-200a;
361506	Leng8	2	1	1	0 rno-miR-141;rno-miR-200a;
362049	Unc5c	2	1	1	0 rno-miR-141;rno-miR-200a;
362092	Brd3	2	1	1	0 rno-miR-141;rno-miR-200a;
362194	Bahd1	2	1	1	0 rno-miR-141;rno-miR-200a;
362485	Ccne2	2	1	1	0 rno-miR-141;rno-miR-200a;
362559	Zyg11b	2	1	1	0 rno-miR-141;rno-miR-200a;
362809	Ptges3	2	1	1	0 rno-miR-141;rno-miR-200a;
362906	Nudcd1	2	1	1	0 rno-miR-141;rno-miR-200a;
363156	Fbxl2	2	1	1	0 rno-miR-141;rno-miR-200a;
363337	LOC363337	2	1	1	0 rno-miR-141;rno-miR-200a;
364033	Vamp4	2	1	1	0 rno-miR-141;rno-miR-200a;
364952	Nkd1	2	1	1	0 rno-miR-141;rno-miR-200a;
365493	Cacul1	2	1	1	0 rno-miR-141;rno-miR-200a;
365703	Zfr	2	1	1	0 rno-miR-141;rno-miR-200a;
365755	Tbl1xr1	2	1	1	0 rno-miR-141;rno-miR-200a;
365903	Fam102b	2	1	1	0 rno-miR-141;rno-miR-200a;
365924	Tmem56	2	1	1	0 rno-miR-141;rno-miR-200a;
366492	Epha2	2	1	1	0 rno-miR-141;rno-miR-200a;
366790	Wibg	2	1	1	0 rno-miR-141;rno-miR-200a;
366894	Trhde	2	1	1	0 rno-miR-141;rno-miR-200a;
366896	Tbc1d15	2	1	1	0 rno-miR-141;rno-miR-200a;
367323	Nudt12	2	1	1	0 rno-miR-141;rno-miR-200a;
497815	Nrcam	2	1	1	0 rno-miR-141;rno-miR-200a;
498003	Dusp3	2	1	1	0 rno-miR-141;rno-miR-200a;
498107	RGD1562339	2	1	1	0 rno-miR-141;rno-miR-200a;
499602	LOC499602	2	1	1	0 rno-miR-141;rno-miR-200a;
500065	Tspan33	2	1	1	0 rno-miR-141;rno-miR-200a;
500941	Msantd4	2	1	1	0 rno-miR-141;rno-miR-200a;
500985	Cbl	2	1	1	0 rno-miR-141;rno-miR-200a;
54284	Pitx2	2	1	1	0 rno-miR-141;rno-miR-200a;
56010	Ywhag	2	1	1	0 rno-miR-141;rno-miR-200a;
56064	Nptn	2	1	1	0 rno-miR-141;rno-miR-200a;
58954	Klf6	2	1	1	0 rno-miR-141;rno-miR-200a;
59265	Phlpp1	2	1	1	0 rno-miR-141;rno-miR-200a;
60325	Serpinb2	2	1	1	0 rno-miR-141;rno-miR-200a;

60586	Clcn4	2	1	1	0	rno-miR-141;rno-miR-200a;
63845	Tmeff1	2	1	1	0	rno-miR-141;rno-miR-200a;
64152	Chp1	2	1	1	0	rno-miR-141;rno-miR-200a;
64189	Pafah1b2	2	1	1	0	rno-miR-141;rno-miR-200a;
64200	Hnrnpf	2	1	1	0	rno-miR-141;rno-miR-200a;
64313	Oat	2	1	1	0	rno-miR-200c;rno-miR-293;
64387	Ccdc80	2	1	1	0	rno-miR-141;rno-miR-200a;
64551	Sept7	2	1	1	0	rno-miR-141;rno-miR-200a;
65190	Rsad2	2	1	1	0	rno-miR-141;rno-miR-200a;
680252	Dmwd	2	1	1	0	rno-miR-141;rno-miR-200a;
680344	Fam189a1	2	1	1	0	rno-miR-141;rno-miR-200a;
681178	Pcgf5	2	1	1	0	rno-miR-141;rno-miR-200a;
681287	Map7d1	2	1	1	0	rno-miR-141;rno-miR-200a;
685232	Atp6v1a	2	1	1	0	rno-miR-141;rno-miR-200a;
685574	LOC685574	2	1	1	0	rno-miR-141;rno-miR-200a;
689174	Zbtb34	2	1	1	0	rno-miR-141;rno-miR-200a;
689844	Maml2	2	1	1	0	rno-miR-141;rno-miR-200a;
690262	Yaf2	2	1	1	0	rno-miR-141;rno-miR-200a;
79433	Myh10	2	1	1	0	rno-miR-141;rno-miR-200a;
81504	Grb2	2	1	1	0	rno-miR-141;rno-miR-200a;
81809	Tgfb2	2	1	1	0	rno-miR-141;rno-miR-200a;
83580	Thbd	2	1	1	0	rno-miR-141;rno-miR-200a;
100125362	LOC100125362	1	1	0	1	rno-miR-200c;
100158233	Slc35a2	1	1	0	1	rno-miR-200c;
100310845	Kdm6a	1	0	1	-1	rno-miR-293;
100360914	Fbxw7	1	1	0	1	rno-miR-200c;
102546648	LOC102546648	1	0	1	-1	rno-miR-293;
102548230	LOC102548230	1	0	1	-1	rno-miR-293;
102556967	LOC102556967	1	0	1	-1	rno-miR-293;
112400	Nrg1	1	1	0	1	rno-miR-200c;
113902	Ces3	1	0	1	-1	rno-miR-293;
113922	Sep15	1	0	1	-1	rno-miR-293;
113992	Ugt1a6	1	0	1	-1	rno-miR-293;
114490	Cited2	1	1	0	1	rno-miR-200c;
114558	Becn1	1	0	1	-1	rno-miR-293;
114856	Dusp1	1	1	0	1	rno-miR-200c;
114906	Pkia	1	1	0	1	rno-miR-200c;
116504	Mprip	1	1	0	1	rno-miR-200c;
116555	Nup107	1	1	0	1	rno-miR-200c;
116744	Lpar1	1	1	0	1	rno-miR-200c;
117268	Khdrbs1	1	1	0	1	rno-miR-200c;
117269	Rps6ka2	1	0	1	-1	rno-miR-293;
117553	Uba3	1	1	0	1	rno-miR-200c;
140666	Rcan2	1	0	1	-1	rno-miR-293;
155140	Il23a	1	0	1	-1	rno-miR-293;
155918	Lcp2	1	1	0	1	rno-miR-200c;
170816	Olr59	1	1	0	1	rno-miR-200c;
170842	Tob1	1	1	0	1	rno-miR-200c;
170914	Nap1l3	1	1	0	1	rno-miR-200c;
171018	Zfp384	1	0	1	-1	rno-miR-200a;
171070	Ptpn21	1	1	0	1	rno-miR-200c;
171396	Sulf1	1	1	0	1	rno-miR-200c;
171410	Acsbg1	1	1	0	1	rno-miR-200c;
192228	Haus1	1	0	1	-1	rno-miR-293;
192235	Hyou1	1	0	1	-1	rno-miR-293;
192270	Ppap2b	1	1	0	1	rno-miR-200c;
24239	Cacna1c	1	1	0	1	rno-miR-200c;
24516	Jun	1	1	0	1	rno-miR-200c;
246074	Scd1	1	1	0	1	rno-miR-200c;
246334	Tp63	1	0	1	-1	rno-miR-293;
24650	Pkd1	1	1	0	1	rno-miR-200c;
24851	Tpm1	1	0	1	-1	rno-miR-293;
25167	Ptpra	1	0	1	-1	rno-miR-200a;
25177	Fhl1	1	1	0	1	rno-miR-200c;
25254	Dspp	1	1	0	1	rno-miR-200c;
25312	Dmp1	1	1	0	1	rno-miR-200c;
25368	Adk	1	1	0	1	rno-miR-200c;
25498	Npm1	1	1	0	1	rno-miR-200c;
25537	Rock2	1	1	0	1	rno-miR-200c;
25573	Ube2i	1	1	0	1	rno-miR-200c;
25603	Marcks	1	1	0	1	rno-miR-200c;
25661	Fn1	1	1	0	1	rno-miR-200c;
266777	Nptx1	1	1	0	1	rno-miR-200c;
266787	Eaf2	1	0	1	-1	rno-miR-293;
26955	Mllt4	1	0	1	-1	rno-miR-293;
288092	Tmem39a	1	0	1	-1	rno-miR-200a;
289080	Rgl1	1	1	0	1	rno-miR-200c;
289178	Dpt	1	0	1	-1	rno-miR-293;
289277	Desi2	1	1	0	1	rno-miR-200c;
289562	RGD1359460	1	1	0	1	rno-miR-200c;
290303	Spryd7	1	1	0	1	rno-miR-200c;
290537	Appl1	1	1	0	1	rno-miR-200c;
290577	Wapal	1	1	0	1	rno-miR-200c;
291078	Prpf4b	1	0	1	-1	rno-miR-293;
291352	Gpr158	1	1	0	1	rno-miR-200c;
29150	Matr3	1	1	0	1	rno-miR-200c;
291671	Hspa9	1	1	0	1	rno-miR-200c;
291863	LOC291863	1	0	1	-1	rno-miR-293;

291914	Cnep1r1	1	1	0	1	rno-miR-200c;
29231	Ciao1	1	0	1	-1	rno-miR-293;
29381	Acvr1b	1	0	1	-1	rno-miR-293;
293849	Zfp275	1	0	1	-1	rno-miR-293;
294518	Sesn1	1	1	0	1	rno-miR-200c;
294732	Actbl2	1	0	1	-1	rno-miR-293;
294790	Skp2	1	1	0	1	rno-miR-200c;
295061	Tm4sf1	1	0	1	-1	rno-miR-293;
29508	Nfya	1	1	0	1	rno-miR-200c;
295395	Rtcd1	1	1	0	1	rno-miR-200c;
295401	Lppr4	1	1	0	1	rno-miR-200c;
29633	Ndst1	1	1	0	1	rno-miR-200c;
296762	Phtf2	1	1	0	1	rno-miR-200c;
297486	Ppp4r2	1	1	0	1	rno-miR-200c;
297801	Xkr4	1	1	0	1	rno-miR-200c;
298757	Atl2	1	1	0	1	rno-miR-200c;
298894	Mycn	1	0	1	-1	rno-miR-293;
299138	Six4	1	0	1	-1	rno-miR-293;
299799	Rab21	1	1	0	1	rno-miR-200c;
299827	Tbk1	1	1	0	1	rno-miR-200c;
299864	Ebag9	1	1	0	1	rno-miR-200c;
300129	Cerk	1	0	1	-1	rno-miR-293;
300289	Amot	1	1	0	1	rno-miR-141;
300756	Arih1	1	1	0	1	rno-miR-200c;
300786	Clpx	1	0	1	-1	rno-miR-293;
300838	Tmod3	1	1	0	1	rno-miR-200c;
301337	Plekhb2	1	0	1	-1	rno-miR-293;
301374	RGD1310553	1	0	1	-1	rno-miR-293;
301513	Rqcd1	1	1	0	1	rno-miR-200c;
301676	Foxn2	1	1	0	1	rno-miR-200c;
302327	Zfp711	1	1	0	1	rno-miR-200c;
302671	Ap1s2	1	1	0	1	rno-miR-200c;
302864	Mmgt1	1	1	0	1	rno-miR-200c;
302920	Rbfox1	1	1	0	1	rno-miR-200c;
303403	Med13	1	0	1	-1	rno-miR-208b-3p;
303439	Mmd	1	1	0	1	rno-miR-200c;
303888	Osbpl11	1	1	0	1	rno-miR-200c;
304023	St3gal6	1	1	0	1	rno-miR-200c;
304294	Rnf216	1	0	1	-1	rno-miR-293;
304301	Mmd2	1	1	0	1	rno-miR-200c;
304546	Git2	1	1	0	1	rno-miR-200c;
304740	Clasp1	1	1	0	1	rno-miR-200c;
304798	Mdm4	1	1	0	1	rno-miR-200c;
304833	Trove2	1	0	1	-1	rno-miR-293;
305104	Col9a1	1	0	1	-1	rno-miR-293;
305127	Zfp644	1	0	1	-1	rno-miR-293;
305131	Lrrc8d	1	0	1	-1	rno-miR-293;
305164	Wdfy3	1	1	0	1	rno-miR-200c;
305230	Shroom3	1	0	1	-1	rno-miR-293;
305268	Uba6	1	1	0	1	rno-miR-200c;
305373	Hmgb3	1	1	0	1	rno-miR-200c;
305968	Hmbox1	1	1	0	1	rno-miR-200c;
306147	Slitrk1	1	1	0	1	rno-miR-200c;
306257	Bap1	1	1	0	1	rno-miR-200c;
306439	Gpm6a	1	1	0	1	rno-miR-200c;
306504	RGD1304810	1	1	0	1	rno-miR-200c;
306862	Tfap2a	1	1	0	1	rno-miR-200c;
307148	Nsun6	1	1	0	1	rno-miR-200c;
307206	Neto1	1	0	1	-1	rno-miR-293;
307362	Zfp532	1	1	0	1	rno-miR-200c;
308306	Eif5b	1	1	0	1	rno-miR-200c;
308942	Dennd5a	1	1	0	1	rno-miR-200c;
309306	RGD1310016	1	1	0	1	rno-miR-200c;
309871	Prdm1	1	1	0	1	rno-miR-200c;
310207	Sema5a	1	0	1	-1	rno-miR-293;
310314	Ttc14	1	1	0	1	rno-miR-200c;
310448	Igsf10	1	1	0	1	rno-miR-200c;
310508	B3galnt1	1	1	0	1	rno-miR-200c;
310533	Rapgef2	1	1	0	1	rno-miR-200c;
310588	Tchh	1	0	1	-1	rno-miR-293;
310846	Tram1l1	1	1	0	1	rno-miR-200c;
311857	Gpr107	1	0	1	-1	rno-miR-293;
312398	Smarcad1	1	1	0	1	rno-miR-200c;
312516	Gmcl1	1	1	0	1	rno-miR-200c;
312562	Zfyve20	1	1	0	1	rno-miR-200c;
312656	March8	1	1	0	1	rno-miR-200c;
312812	Eps8	1	1	0	1	rno-miR-200c;
313033	Xkr8	1	1	0	1	rno-miR-200c;
313370	Hook1	1	1	0	1	rno-miR-200c;
313413	Sgip1	1	1	0	1	rno-miR-200c;
313488	Reck	1	1	0	1	rno-miR-200c;
313609	Pdik1l	1	1	0	1	rno-miR-200c;
313620	Srrm1	1	0	1	-1	rno-miR-293;
313729	Errfi1	1	1	0	1	rno-miR-200c;
313811	Dnajb5	1	1	0	1	rno-miR-200c;
313997	Mboat2	1	1	0	1	rno-miR-200c;
314157	Fbxo33	1	1	0	1	rno-miR-200c;
314548	Osgepl1	1	1	0	1	rno-miR-200c;

314630	Rexo1	1	0	1	-1	rno-miR-293;
314930	Zfpm2	1	1	0	1	rno-miR-200c;
314942	Csmd3	1	1	0	1	rno-miR-200c;
315496	Dpy19l1	1	1	0	1	rno-miR-200c;
315549	Fam118b	1	1	0	1	rno-miR-200c;
315891	RGD1309079	1	1	0	1	rno-miR-200c;
316021	Epm2aip1	1	1	0	1	rno-miR-200c;
316237	Mad2l1bp	1	1	0	1	rno-miR-200c;
316344	Rev1	1	1	0	1	rno-miR-200c;
317439	Wwc3	1	1	0	1	rno-miR-200c;
360272	Slit2	1	1	0	1	rno-miR-200c;
361089	Ndfip2	1	1	0	1	rno-miR-200c;
361104	Zcchc24	1	1	0	1	rno-miR-200c;
361171	Golga7	1	1	0	1	rno-miR-200c;
361351	Hdhd2	1	1	0	1	rno-miR-200c;
361367	Amfr	1	1	0	1	rno-miR-200c;
361626	Tmem41b	1	1	0	1	rno-miR-200c;
361814	Srsf3	1	0	1	-1	rno-miR-293;
362154	Zc3h15	1	1	0	1	rno-miR-200c;
362261	Lpin3	1	1	0	1	rno-miR-200c;
362279	Pard6b	1	1	0	1	rno-miR-200c;
362304	Orc5	1	0	1	-1	rno-miR-293;
362325	Mdfic	1	1	0	1	rno-miR-200c;
362521	Tmem38b	1	1	0	1	rno-miR-200c;
362578	Ccdc23	1	0	1	-1	rno-miR-293;
362700	Spast	1	0	1	-1	rno-miR-293;
362728	Twistnb	1	1	0	1	rno-miR-200c;
362733	Etv1	1	0	1	-1	rno-miR-293;
362750	Atl1	1	1	0	1	rno-miR-141;
362859	Ckap4	1	1	0	1	rno-miR-200c;
363062	Dixdc1	1	0	1	-1	rno-miR-293;
363089	Fam63b	1	1	0	1	rno-miR-200c;
363231	Rftn2	1	1	0	1	rno-miR-200c;
363266	Agfg1	1	1	0	1	rno-miR-200c;
363455	Chrdl1	1	1	0	1	rno-miR-200c;
363463	Mospd2	1	1	0	1	rno-miR-200c;
364634	Csmd1	1	0	1	-1	rno-miR-293;
364686	Ranbp9	1	1	0	1	rno-miR-200c;
365894	Trim33	1	1	0	1	rno-miR-200c;
366065	Cers6	1	1	0	1	rno-miR-200c;
366300	Pcmtd1	1	0	1	-1	rno-miR-293;
366734	Bag5	1	1	0	1	rno-miR-200c;
373544	Ermp1	1	1	0	1	rno-miR-200c;
432358	Elavl4	1	1	0	1	rno-miR-141;
497961	Nlk	1	0	1	-1	rno-miR-208b-3p;
497967	Znf830	1	0	1	-1	rno-miR-293;
498000	Coa3	1	0	1	-1	rno-miR-293;
498266	Blzf1	1	1	0	1	rno-miR-200c;
498331	Ptpn13	1	1	0	1	rno-miR-200c;
499129	Kctd15	1	1	0	1	rno-miR-200c;
499583	Naaladl2	1	1	0	1	rno-miR-200c;
499615	Lhfp	1	1	0	1	rno-miR-200c;
499624	Tsc22d2	1	1	0	1	rno-miR-200c;
500069	Tsga14	1	1	0	1	rno-miR-200c;
500551	S100pbp	1	1	0	1	rno-miR-200c;
500651	Sptssa	1	1	0	1	rno-miR-200c;
501559	RGD1562161	1	1	0	1	rno-miR-200c;
502776	Scrn1	1	0	1	-1	rno-miR-293;
503027	Map4k5	1	1	0	1	rno-miR-200c;
503052	Vash1	1	1	0	1	rno-miR-200c;
50555	Ugt8	1	1	0	1	rno-miR-200c;
50572	Slco1a1	1	1	0	1	rno-miR-200c;
50621	Slc23a1	1	1	0	1	rno-miR-200c;
50658	Mapk9	1	1	0	1	rno-miR-200c;
54230	Btg3	1	0	1	-1	rno-miR-293;
54254	Gata4	1	1	0	1	rno-miR-200c;
64525	Tceb1	1	1	0	1	rno-miR-200c;
64823	Csnk1g3	1	1	0	1	rno-miR-200c;
65180	Kcnd2	1	1	0	1	rno-miR-200c;
680039	LOC680039	1	0	1	-1	rno-miR-293;
685009	Rnf169	1	1	0	1	rno-miR-200c;
685790	LOC685790	1	1	0	1	rno-miR-200c;
688581	Elmod2	1	1	0	1	rno-miR-200c;
688843	Nap1l5	1	1	0	1	rno-miR-200c;
689079	Arl15	1	1	0	1	rno-miR-200c;
689890	Srsf1	1	1	0	1	rno-miR-200c;
690043	Rnf168	1	1	0	1	rno-miR-200c;
691517	Megf11	1	1	0	1	rno-miR-200c;
79212	Slc6a1	1	1	0	1	rno-miR-200c;
79224	Serpind1	1	0	1	-1	rno-miR-293;
81636	Adcy2	1	1	0	1	rno-miR-200c;
81652	Ctbs	1	1	0	1	rno-miR-200c;
81748	Pls3	1	1	0	1	rno-miR-200c;
83469	Lrp4	1	1	0	1	rno-miR-200c;
83785	Vegfa	1	1	0	1	rno-miR-200c;
83840	Rps6kb1	1	1	0	1	rno-miR-200c;
84030	Chn1	1	1	0	1	rno-miR-200c;
84407	Cdh11	1	1	0	1	rno-miR-200c;

84409	Robo2	1	1	0	1	rno-miR-200c;
84481	Arid4b	1	1	0	1	rno-miR-200c;
84587	Plcl1	1	1	0	1	rno-miR-200c;
84686	Ppp1r9b	1	1	0	1	rno-miR-200c;
94267	Nudt4	1	1	0	1	rno-miR-200c;
94269	Fez2	1	1	0	1	rno-miR-200c;
94273	Mgat2	1	1	0	1	rno-miR-200c;
